# Supplementary material for: The Late Quaternary climate impact on the genome of the woodland strawberry (Fragaria vesca), a perennial herb
Source: Commun Biol. 2026 Jan 15;9:263. doi: 10.1038/s42003-026-09539-5 (PMC12913768; doi:10.1038/s42003-026-09539-5)
Supplement: Supplementary file 2 — Supplementary information [file 42003_2026_9539_MOESM2_ESM.pdf]

The Late Quaternary climate impact on the genome of the woodland strawberry (*Fragaria vesca*), a perennial herb

Tuomas Toivainen, J. Sakari Salonen, Jonathan Kirshner, Sergei Lembinen, Hanne De Kort, Annina Lyyski, Patrick P. Edger, Hrannar Smári Hilmarsson, Jón Hallsteinn Hallsson, Daniel J. Sargent, Klaus Olbricht, José F. Sánchez-Sevilla, Laura Jaakola, Johan A. Stenberg, Boris Duralija, Juozas Labokas, Henry Väre, Jarkko Salojärvi, Petri Auvinen, David Posé, Victor A. Albert, Timo Hytönen

Corresponding author:
Prof. Timo Hytönen
Email: timo.hytonen@helsinki.fi

This PDF file includes:

Supplementary Note 1 and 2
Supplementary Figure 1-26
Supplementary Table 1

Supplementary Note 1. High resolution MSMC-IM analysis in samples with low F_{ROH}

For historical effective population size (N_E) and migration rates (m), we focused on common clear patterns (Supplementary Fig. 17), that aligned with the chronological timing of serial MIS[1]. Combinations of samples with low F_{ROH} (e.g. core-core) consistently yielded high temporal resolution demographic histories (Fig. 3A, B), whereas in peripheral regions, samples with the lowest F_{ROH} (Supplementary Data 4; Supplementary Fig. 25) provided often accurate temporal alignment with Marine Isotope Stages (1) when combined with a sample with low F_{ROH} (source population) (Fig. 3C-G, Supplementary Fig. 19). In contrary, MSMC-IM did not work successfully without extreme parameter fluctuations when both N1 and N2 samples were small and highly inbred (high F_{ROH}). Demographic history patterns across all runs were divided into different categories according to the following criteria (Supplementary Fig. 17, Supplementary Data 4): CORE=Stable migration rate from MIS 10 until the Last Glacial Maximum (LGM), slightly reduced Ne during PGP; PERIPHERAL-1=One strong bottleneck in addition to the LGM bottleneck during either Penultimate Glacial Period (PGP) or MIS 8, fluctuating migration rate at least during two G-IG cycles between PGP and MIS 10; PERIPHERAL-2=Stable N_E, migration completely terminated at least once between MIS 10 and LGM; PERIPHERAL-3=One strong bottleneck and termination of migration rate at least once between MIS 10 and LGM; PERIPHERAL-4=Strong bottleneck between MIS 10 and PGP, no termination of migration between MIS 10 and LGM. In the figures (Fig. 3, Supplementary Fig. 21), we present the results of CORE and PERIPHERAL-1 patterns using the samples with the lowest F_{ROH} values across regions (primary data). Results of all MSMC-IM runs are available in Supplementary Data 4 and the proportions of different patterns visually shown in Supplementary Fig. 17.

Supplementary Note 2. Reliability of migration rates

In general, migration rate provided higher resolution in detecting glacial periods (up to four glaciations identified as complete cessations of migration) compared with the traditionally used effective population size (N_E; up to two glaciations detected as strong bottlenecks), possibly because N_1 and N_2 estimates tended to converge in the distant past. In MSMC-IM runs, sample pairs with a cumulative migration probability (M) lower than 0.999 (Supplementary Fig. 21), a threshold deemed acceptable for detecting migration rates between two ancestral lineages according to Wang et al. (2020) (2), exhibited migration patterns identical to those above the threshold (M>0.999, Supplementary Fig. 21). Specifically, the peripheral pattern showed a complete cessation of migration during MIS 10, MIS 8, MIS 6 (PERIPHERAL 1) glacial periods and more recently during the MIS 2. There can be several reasons for that. In the original paper in humans (2), the maximum age of initial divergence (M<0.999) was about 60 000 generations, after which two lineages were fully randomized. In this study, we found consistent patterns for up to 165,000-185,000 generations (330-370 ka, corresponding to MIS 10 in the peripheral pattern, indicating a deeper signal in woodland strawberry compared to humans. Consistent with this, several population pairs began to diverge (M < 0.999) during MIS 10 (Supplementary Fig. 15). For example, four independent sample pairs between Iberia and Italy (different refugia) started to diverge (M<0.999) during that time. In the core pattern, distant initial divergence events (M<0.999) occurred less frequently, with only a few population pairs between Italy/Croatia and Romania or Lithuania and Romania, initiating divergence during or before MIS10. Consequently, there is greater uncertainty regarding the migration rates in the core pattern. In general, their divergence occurs more recently compared to peripheral pattern (Supplementary Fig. 15), possibly indicating that they originate from a large common eastern ancestral population.

Both genomic and biological reasons can contribute to this difference between strawberries and humans. In small genomes like in woodland strawberry (~220Mb), where genic regions comprise nearly half of the genome, accurate haplotype reconstruction is relatively straightforward compared with larger, more repetitive genomes like that of humans. During glaciations, woodland strawberries, particularly those in peripheral refugia, were likely forced into predominantly autogamous reproduction, as our results suggest for present-day peripheral populations. This transition would have led to complete cessation of migration between refugia. Based on observed migration rate patterns (Fig. 3B, D, F; Supplementary Fig. 12-14), following glacial retreat, these lineages came into secondary contact during interglacial range expansions - either with large,

genetically diverse source populations or with other formerly autogamous refugial lineages. It should be noted that the number of refugia that existed during glaciations, beyond the western (i.e., Iberian and Apennine) and eastern (i.e., Balkan) southern refugia, remains unknown. We found evidence for both western (Fig. 1C) and eastern (i.e., Alta and Kåfjord) microrefugia that may have existed during earlier glaciations, although their precise historical geographic locations cannot be inferred from the current locations of populations. In any case, it is possible that the varying time-dependent modes of reproduction and cycling between ancestral homozygosity and heterozygosity are more readily detectable in strawberry genomes than in human genomes, where self-fertilization does not occur. Extensive recontact events, particularly during the Holocene, are evident in strawberry genomes (Fig. 3B, D, F, Supplementary Fig. 11-14). Sample pairs showing less recontact during the Holocene, such as between Iberia and Lithuania/Romania, which split during the MIS2, began diverging ($M<0.999$) consistently in a deeper past (Supplementary Fig. 15). This supports our hypothesis that extensive secondary contacts during the Holocene have often increased the cumulative migration probability threshold above 0.999, despite the possibility that old parts of ancestral lineages can still be distinguishable. These factors suggest that studies on strawberry genomes might reveal deeper initial divergences ($M>0.999$) compared to those observed in human populations.

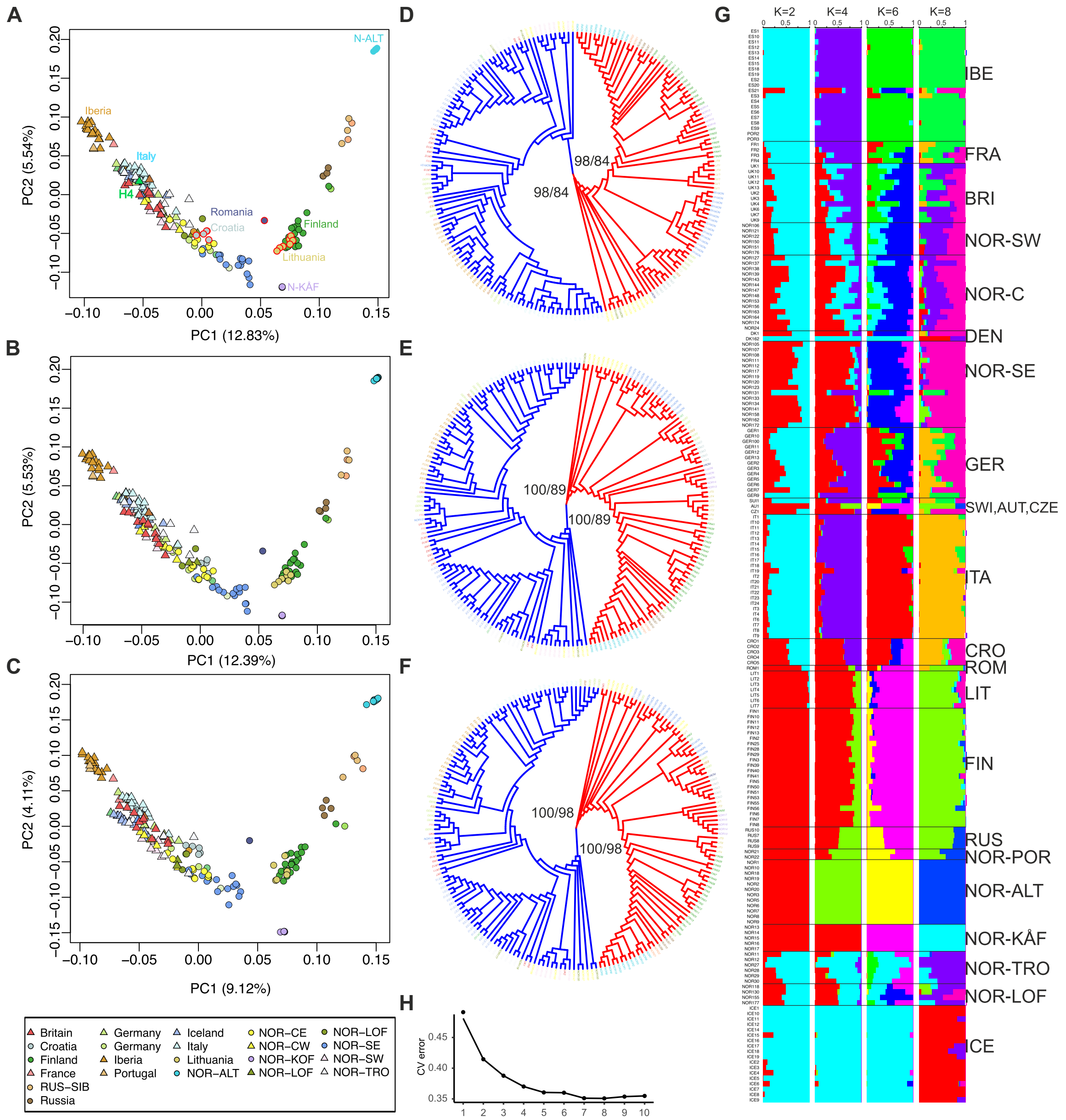

Supplementary Figure 1. Population structure using different genomic datasets and analyses. A-C) PCA using (A) 4-fold degenerate sites, (B) nonsynonymous sites and (C) LD-pruned set of SNPs across the whole genome. D-F) Maximum likelihood phylogenetic analysis based on corresponding genomic regions. Bootstrap support values (SH-aLRT/ultrafast) for western and eastern branch are shown (for all branches in Dataset S02). G) Admixture plot with K=2, K=4, K=6 and K=8 using 4-fold degenerate sites. H) Cross-validation error for different values of K. Abbreviations: IBE=Iberia, FRA=France, BRI=Britain, NOR-SW=Norway-southwestern, NOR-C=Norway-central, DEN=Denmark, NOR-SE=Norway-southeastern, GER=Germany, SWI=Switzerland, AUT=Austria, CZE=Czechia, ITA=Italy, CRO=Croatia, ROM=Romania, LIT=Lithuania, FIN=Finland, RUS=Russia, NOR-POR=Norway-Porsanger, NOR-ALT=Norway-Alta, NOR-KÅF=Norway-Kåfjord, NOR-TRO=Norway-Tromso, NOR-LOF=Norway-Lofoten, ICE=Iceland.

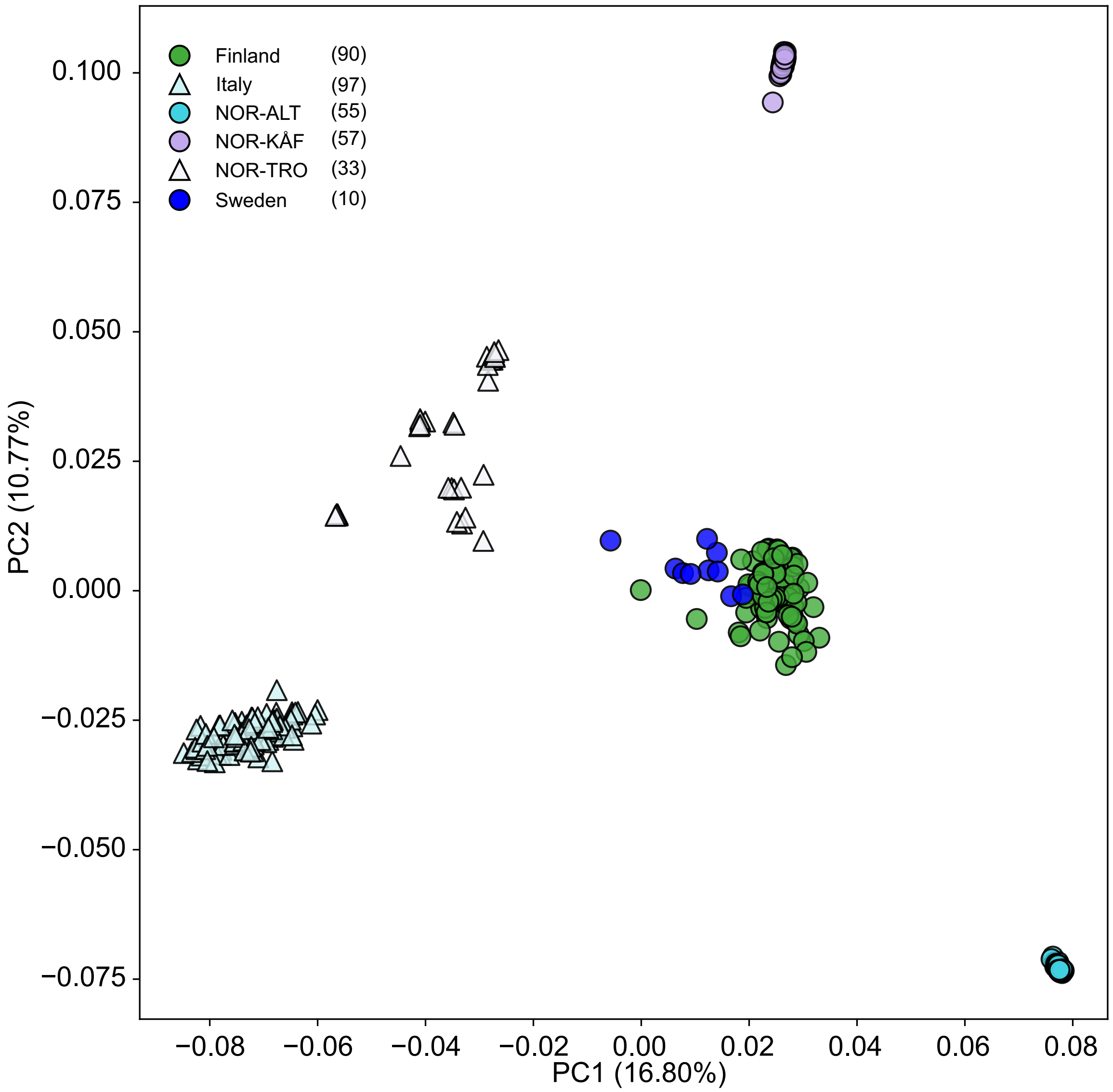

Supplementary Figure 2. Population structure of genotyping by sequencing (GBS) data. Principal component (PC) analysis using all SNPs. Number of sequenced samples from each region are shown in parentheses. Abbreviations: NOR-ALT= Norway-Alta, NOR-KÅF= Norway-Kåfjord, NOR-TRO= Norway-Tromsø

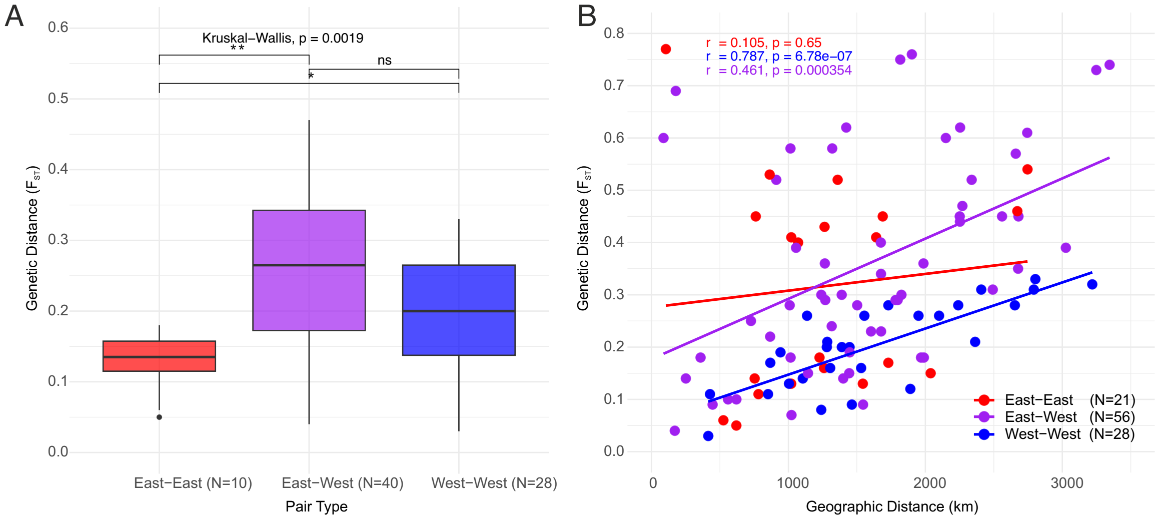

Supplementary Figure 3. Genetic differentiation and isolation by distance (IBD). A) Genetic differentiation (F_{ST}) between regions across three different pair types excluding highly differentiated Alta (NOR-ALT) and Kåfjord (NOR-KÅF) samples. Regions are categorized as western or eastern if more than half of samples belong to the western or eastern cluster, respectively. Statistical significance between pairwise comparisons shown. Note that we expect eastern regions having lower F_{ST} due to shorter geographic distances between the regions (Fig.1E). B) IBD across all regions including northern Norwegian Alta and Kåfjord samples. Correlation coefficients shown with statistical significances by cor.test function in R. Number of region pairs for each category are shown in parentheses.

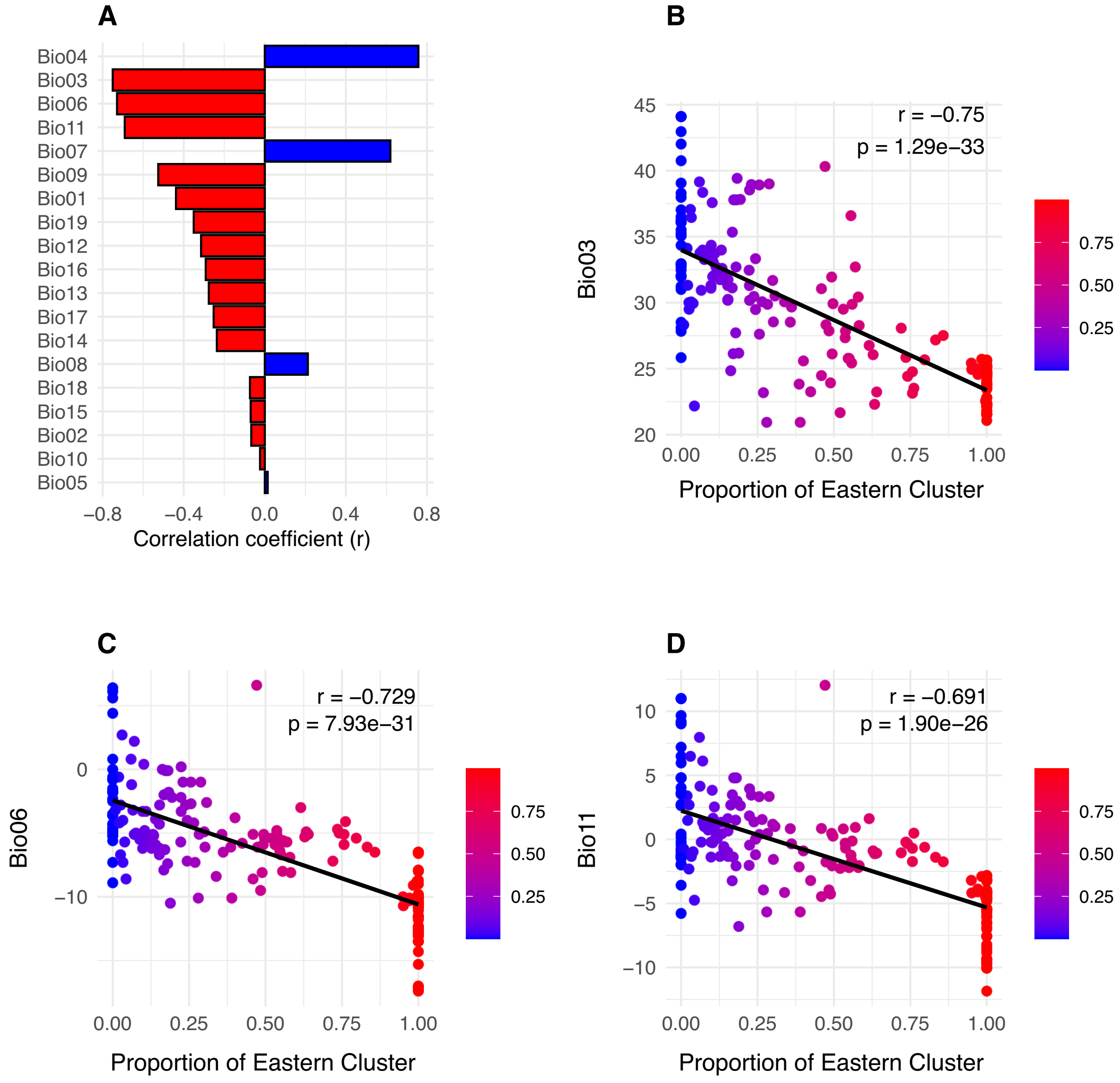

Supplementary Figure 4. Correlation of bioclimatic variables and the genetic clustering of samples. A) Correlation coefficients between the eastern admixture proportion of the genome and 19 bioclimatic variables across samples (N=185 or 186 depending on bioclimatic variable), sorted by absolute values. B-D) The correlations of bioclimatic variables Bio3, Bio6 and Bio11 with the admixture proportions. The strongest correlation (Bio4) is shown in Fig. 1F. Pearson correlation coefficients with Bonferroni-corrected p-values, calculated using the p.adjust function in R, are shown. Bio1 = Annual Mean Temperature, Bio2= Mean Diurnal Range (Mean of monthly (max temp - min temp)), Bio3= Isothermality (Bio2/Bio7) × 100, Bio4 =Temperature Seasonality (standard deviation × 100), Bio5 = Max Temperature of Warmest Month, Bio6= Min Temperature of Coldest Month, Bio7 = Temperature Annual Range (Bio5–Bio6), Bio8=Mean Temperature of Wettest Quarter, Bio9 = Mean Temperature of Driest Quarter, Bio10= Mean Temperature of Warmest Quarter, Bio11= Mean Temperature of Coldest Quarter, Bio12 = Annual Precipitation, Bio13=Precipitation of Wettest Month, Bio14= Precipitation of Driest Month, Bio15= Precipitation Seasonality (Coefficient of Variation), Bio16= Precipitation of Wettest Quarter, Bio17= Precipitation of Driest Quarter, Bio18 = Precipitation of Warmest Quarter, Bio19= Precipitation of Coldest Quarter.

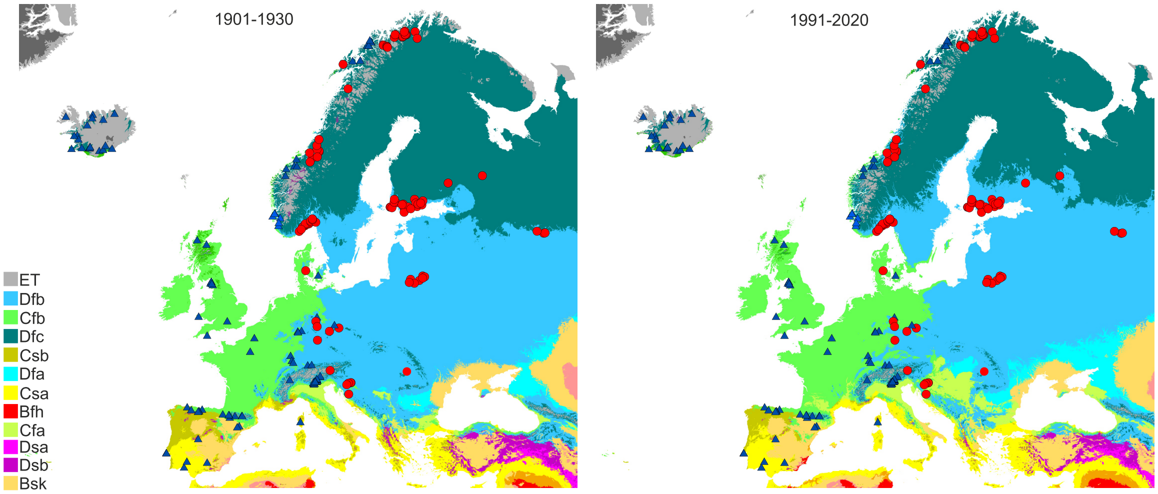

Supplementary Figure 5. Geographical division of the western (N=107) and eastern (N=92) genetic clusters of woodland strawberry (based on maximum likelihood clustering at 4-fold degenerate sites) follows the border separating oceanic (Cfb) and continental (Dfb) or sub-arctic (Dfc) climatic zones across Europe (Beck et al. 2023). Modeling the distributions of climatic zones for two different time-intervals: A) (1901-1930) and B) (1991-2020). Other climatic zones: ET=Tundra, Csb=Warm-summer Mediterranean climate, Dfa=Hot-summer humid continental climate, Csa=Hot-summer Mediterranean climate, Bfh=Hot desert climate, Cfa=Humid subtropical climate, Dsa = Mediterranean-influenced hot-summer humid continental climate, Dsb = Mediterranean-influenced warm-summer humid continental climate, BSk = Cold semi-arid climate. Data were visualized with ArcMap-software.

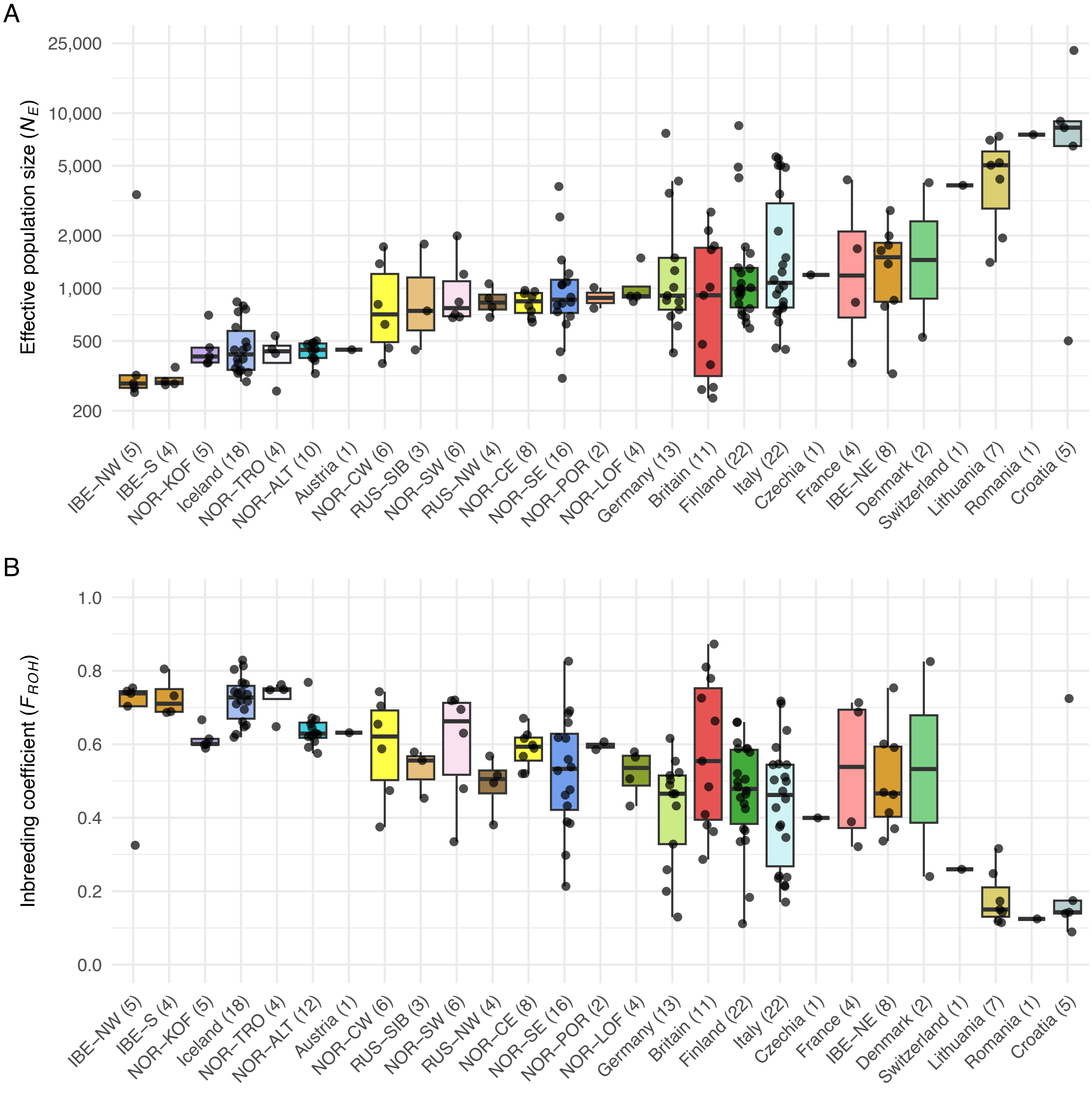

Supplementary Figure 6. Effective population size (A) and regional inbreeding coefficients (F_{ROH}) based on runs of homozygosity (ROH) (B) ordered by increasing N_E. Numbers at the bottom of figures show the number of samples used in corresponding figures. Abbreviations: IBE-S= Iberia-southern, IBE-NW= Iberia-northwestern, NOR-TRO=Norway-Tromsø, NOR-KÅF=Norway-Kåfjord, NOR-POR=Norway-Porsanger, NOR-ALT=Norway-Alta, NOR-CW=Norway-central-western, RUS-SIB=Russia-Siberia, Russia-NW=Russia-northwestern, Karelia=Russia-Karelia, NOR-SE=Norway-southeastern, NOR-LOF=Norway-Lofoten, NOR-SW=Norway-southwestern, NOR-CE=Norway-central-eastern, IBE-NE=Iberia-northeastern.

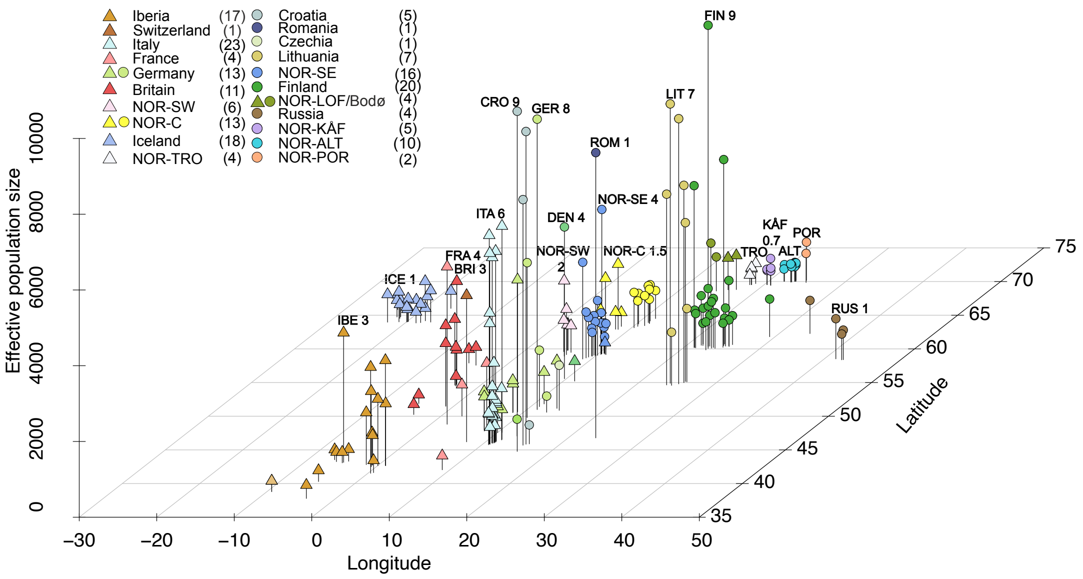

Supplementary Figure 7. Populations at the edges of the range have the lowest effective population sizes (N_E). N_E-values plotted with geographic coordinates of each sample. Population with the highest N_E in each region is highlighted.

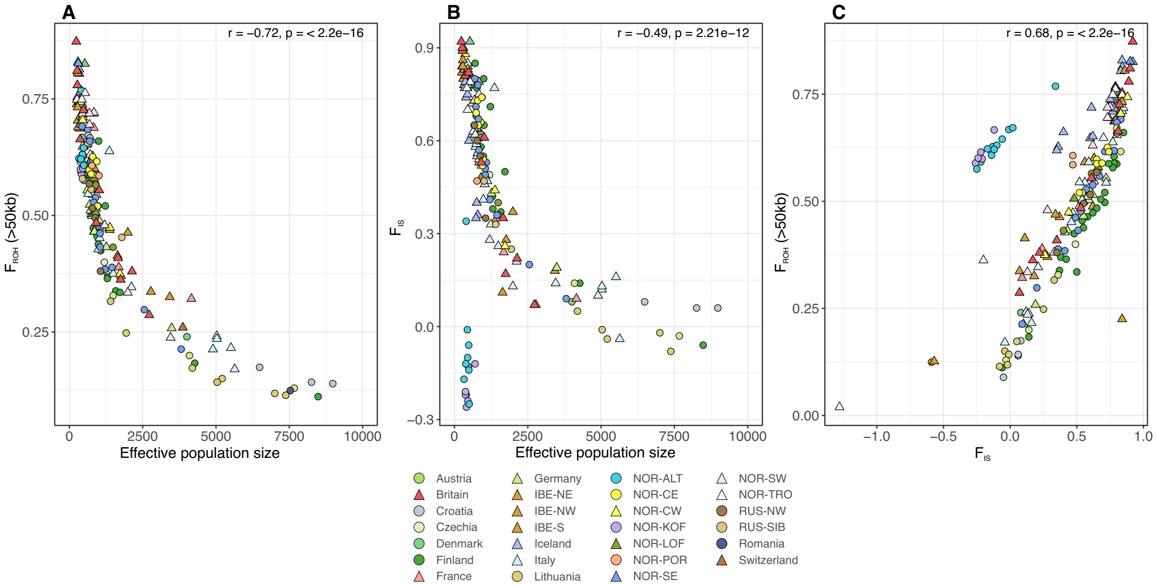

Supplementary Figure 8. Correlation of inbreeding coefficients and present effective population sizes (N_E). A) Correlation between N_E and inbreeding coefficient based on Runs of Homozygosity (F_{ROH}). B) Correlation between N_E and inbreeding coefficients based on regional inbreeding coefficients (F_{IS}) and C) correlation between F_{IS} and F_{ROH} including outlier samples.

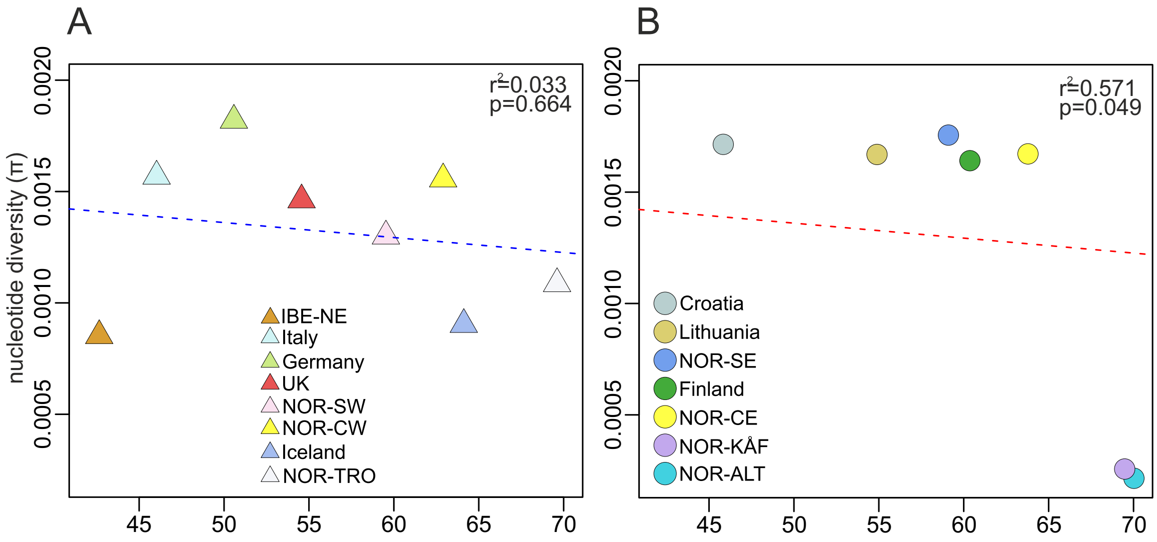

Supplementary Figure 9. Latitudinal correlation of genetic diversity. Intergenic nucleotide diversity (π) per region in A) western and B) eastern Europe. The coefficient of determination (r²) is derived from the Pearson correlation coefficient (r), with statistical significance indicated by the *p*-value.

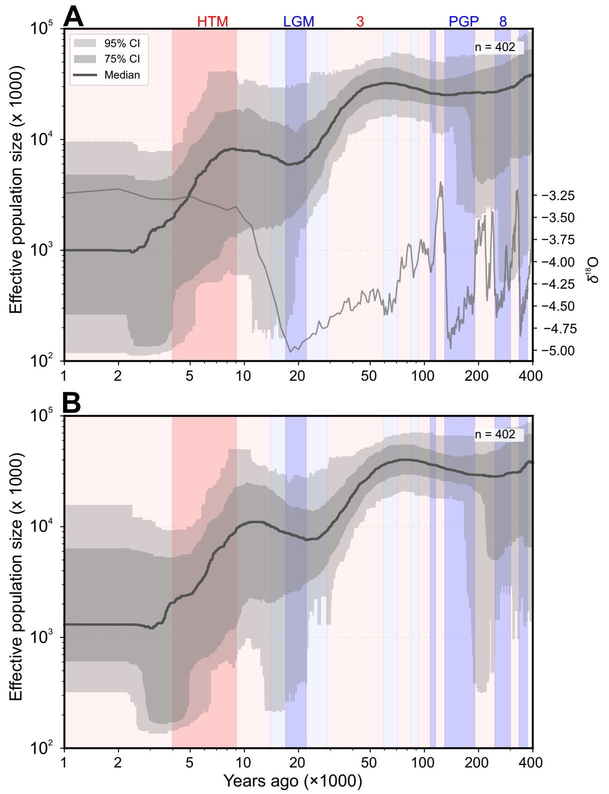

Supplementary Figure 10. Demographic history of woodland strawberry in all samples using two different mutation rates. A, B) Effective population size using the mutation rate of *Arabidopsis thaliana* (A) and *Fragaria* genus (B). C, D) Migration rate using the mutation rate of *Arabidopsis thaliana* (C) and *Fragaria* genus. Medians and confidence intervals (CIs) were calculated from the distribution of biological replicates using the empirical percentile method, following Wang et al. (2020). Both 95% and 75% CIs were derived from the corresponding percentiles, with values for migration rates estimated on the log scale (n=sample size). HTM=Holocene Thermal Maximum (9,000-4,000 ya), LGM=Last Glacial Maximum (22,000-17,000 ya), 3= Marine isotope stage 3, PGP=Penultimate Glacial Period (190,000-130,000 ya), 8=Marine isotope stage 8. Gray curve in A represent inverse benthic δ18O records from Lisiecki and Raymo (2005)[1] and is used as a proxy for historical temperature.

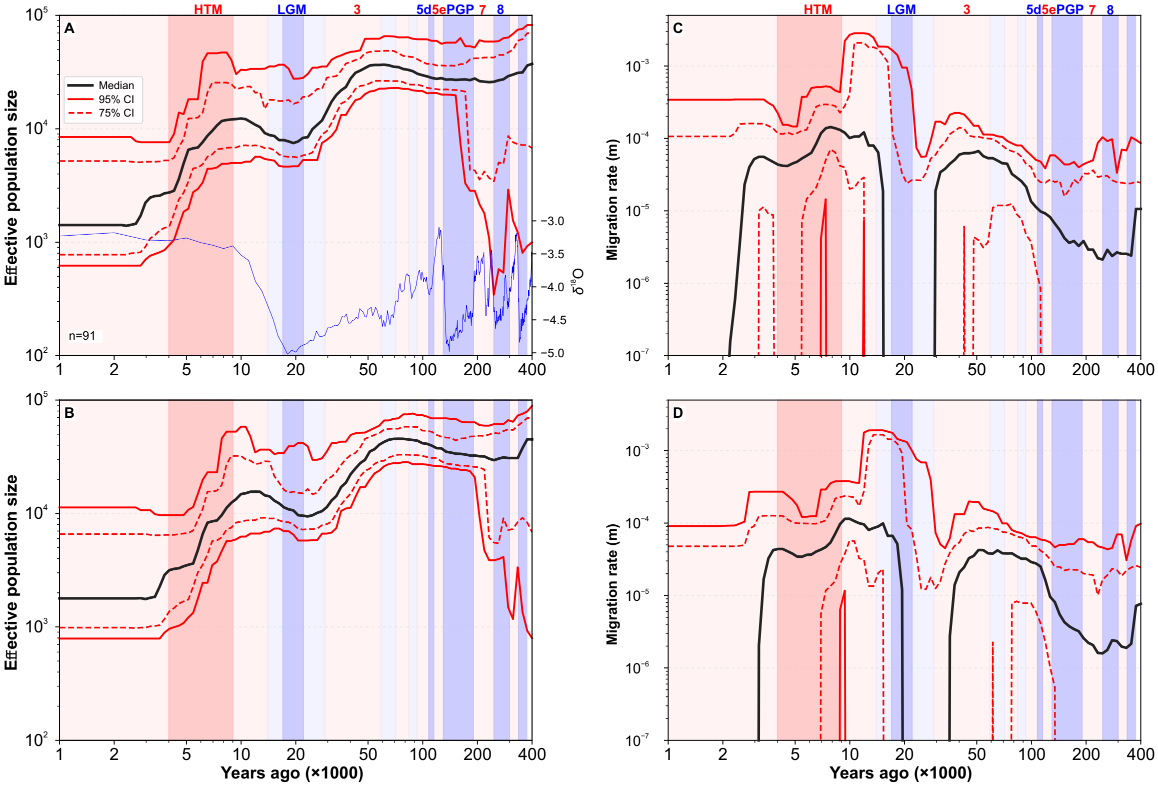

Supplementary Figure 11. Demographic history of woodland strawberry inferred using two mutation rates. A-D) Effective population sizes (A, B) and migration rates (C, D) using the mutation rate of *Arabidopsis thaliana* (A,C) or the *Fragaria* genus (B,D). Medians and confidence intervals (CIs) were calculated from the distribution of biological replicates using the empirical percentile method (n=91), following Wang et al. (2020). Both 95% and 75% CIs were derived from the corresponding percentiles, with migration rate values estimated on the log scale. The runs drawn where isolation event midpoints fall within the MIS 2 glaciation (29–14 kya) when using the *Arabidopsis* mutation rate and show the isolation event shifted 26.7% later when using the *Fragaria* mutation rate, consistent with the expected difference between species' mutation rates. HTM = Holocene Thermal Maximum (9,000–4,000 ya); LGM = Last Glacial Maximum (22,000–17,000 ya); PGP = Penultimate Glacial Period (190,000–130,000 ya). Marine Isotope Stages (MIS) are indicated by numbers. Blue curve in A represent inverse benthic δ18O records from Lisiecki and Raymo (2005)[1] and serves as a proxy for historical temperature.

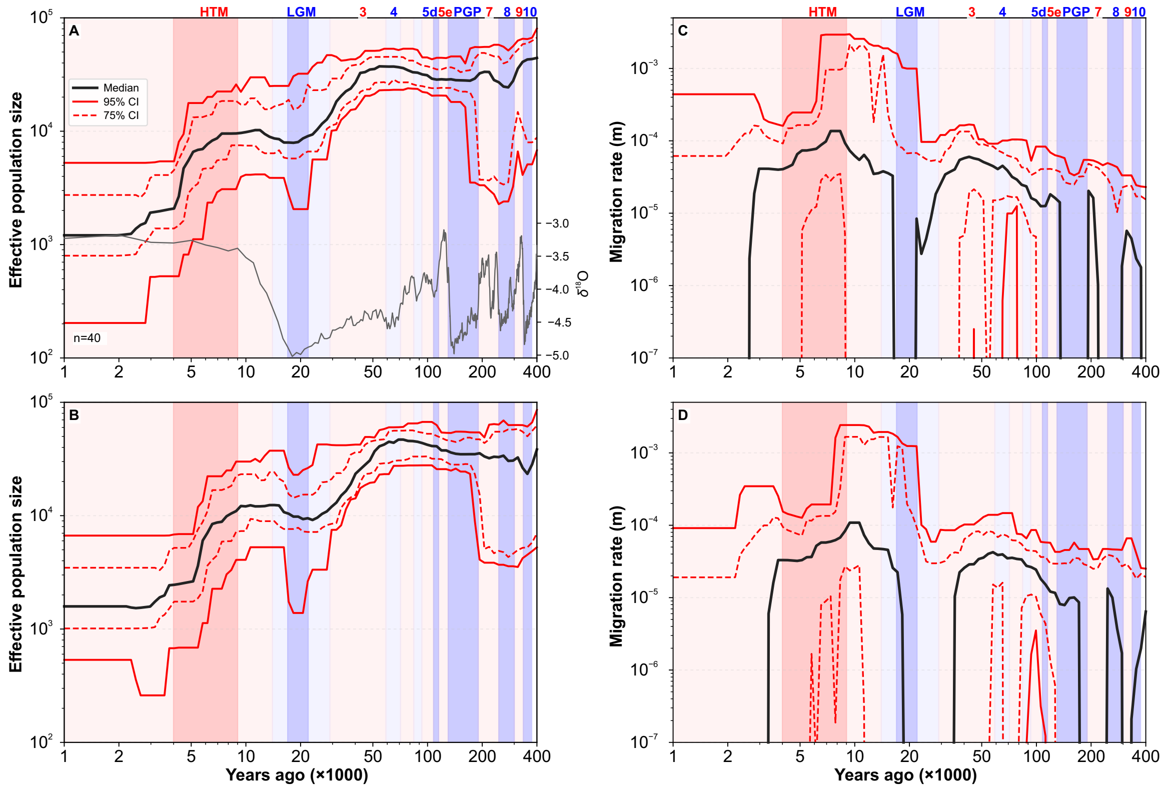

Supplementary Figure 12. Demographic history (Peripheral-1) of woodland strawberry inferred using two mutation rates. A-D) Effective population sizes (A, B) and migration rates (C, D) using the mutation rate of *Arabidopsis thaliana* (A,C) or the *Fragaria* genus (B,D). Medians and confidence intervals (CIs) were calculated from the distribution of biological replicates using the empirical percentile method (n = 40), following Wang et al. (2020). Both 95% and 75% CIs were derived from the corresponding percentiles, with migration rate values estimated on the log scale. The runs shown have isolation event midpoints falling within the Penultimate Glacial Period (PGP; 190–130 kya) when using the *Arabidopsis* mutation rate, while the use of the *Fragaria* mutation rate results in the expected shift of 26.7% in the midpoint of the corresponding isolation event. HTM = Holocene Thermal Maximum (9,000–4,000 ya); LGM = Last Glacial Maximum (22,000–17,000 ya); PGP = Penultimate Glacial Period (190,000–130,000 ya). Marine Isotope Stages (MIS) are indicated by numbers. Gray curve in A represent inverse benthic δ18O records from Lisiecki and Raymo (2005)[1] and serves as a proxy for historical temperature.

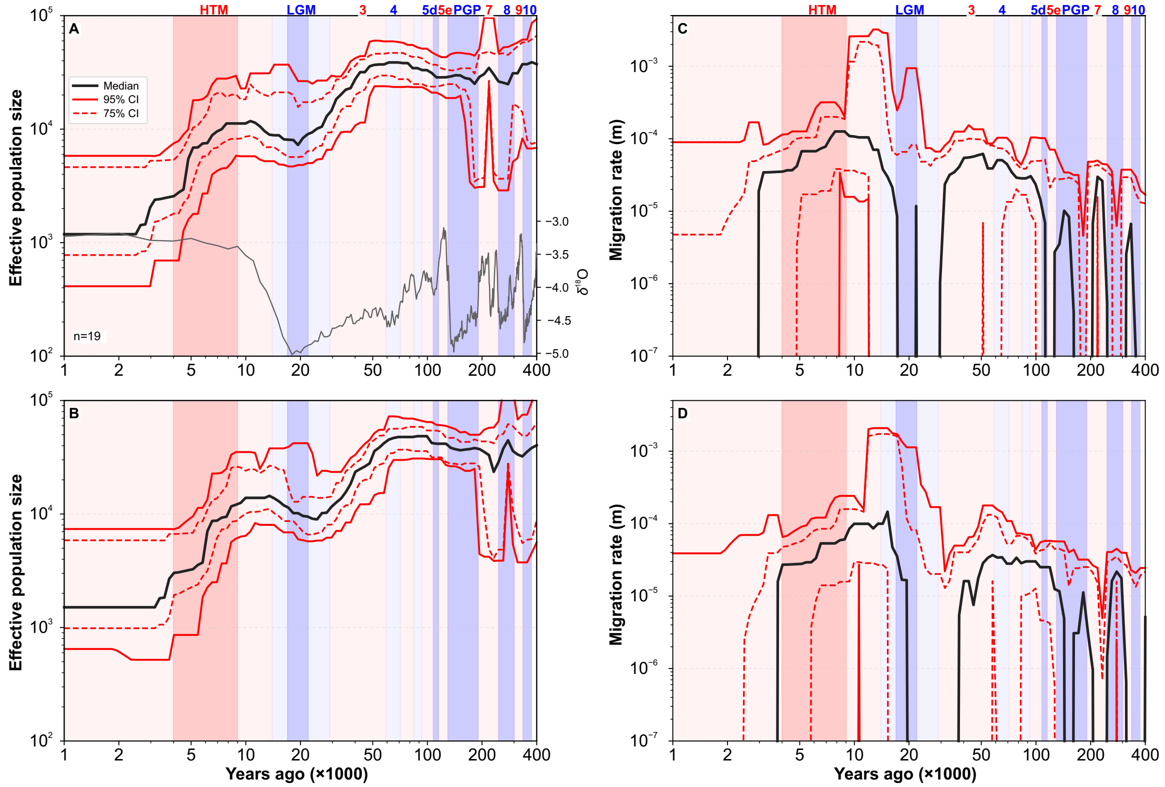

Supplementary Figure 13. Demographic history (Peripheral-1) of woodland strawberry inferred using two mutation rates. A-D) Effective population sizes (A, B) and migration rates (C, D) using the mutation rate of *Arabidopsis thaliana* (A, C) or the *Fragaria* genus (B, D). Medians and confidence intervals (CIs) were calculated from the distribution of biological replicates using the empirical percentile method (n = 19), following Wang et al. (2020). Both 95% and 75% CIs were derived from the corresponding percentiles, with migration rate values estimated on the log scale. The runs shown have isolation event midpoints falling within the MIS8 glaciation (300–243 kya) when using the *Arabidopsis* mutation rate, while the use of the *Fragaria* mutation rate results in the expected shift of 26.7% in the midpoint of the corresponding isolation event. HTM = Holocene Thermal Maximum (9,000–4,000 ya); LGM = Last Glacial Maximum (22,000–17,000 ya); PGP = Penultimate Glacial Period (190,000–130,000 ya). Marine Isotope Stages (MIS) are indicated by numbers. Gray curve in A represent inverse benthic δ18O records from Lisiecki and Raymo (2005)[1] and serves as a proxy for historical temperature.

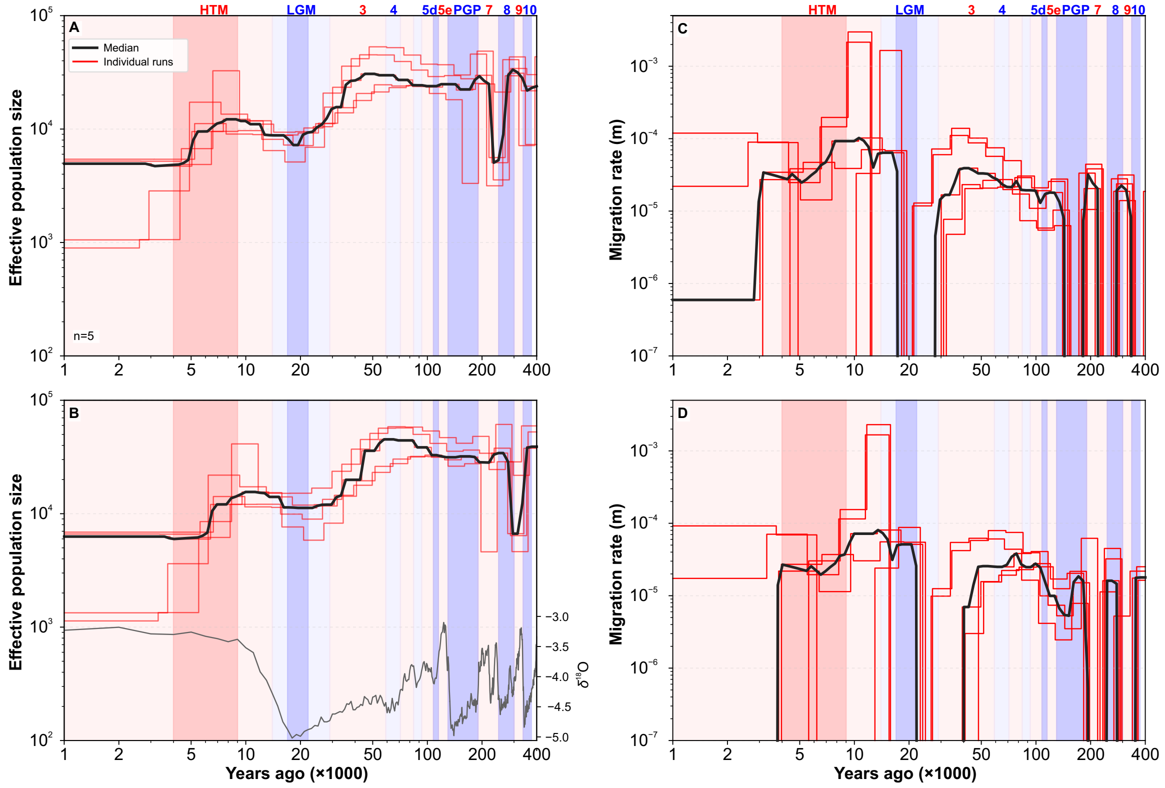

Supplementary Figure 14. Demographic history (Peripheral-1) of woodland strawberry inferred using two mutation rates (n = sample size). A-D) Effective population sizes (A,B) and migration rates (C,D) using the mutation rate of *Arabidopsis thaliana* (A,C) or the *Fragaria* genus (B,D). Medians calculated from individual runs, CIs were not determined due to small sample size (n = 5). The runs shown have isolation event midpoints falling within the MIS10 glaciation (375–335 kya) when using the *Arabidopsis* mutation rate, while the use of the *Fragaria* mutation rate results in the expected shift of 26.7% in the midpoint of the corresponding isolation event. HTM = Holocene Thermal Maximum (9,000–4,000 ya); LGM = Last Glacial Maximum (22,000–17,000 ya); PGP = Penultimate Glacial Period (190,000–130,000 ya). Marine Isotope Stages (MIS) are indicated by numbers. Gray curve in B represent inverse benthic δ18O records from Lisiecki and Raymo (2005)[1] and serves as a proxy for historical temperature.

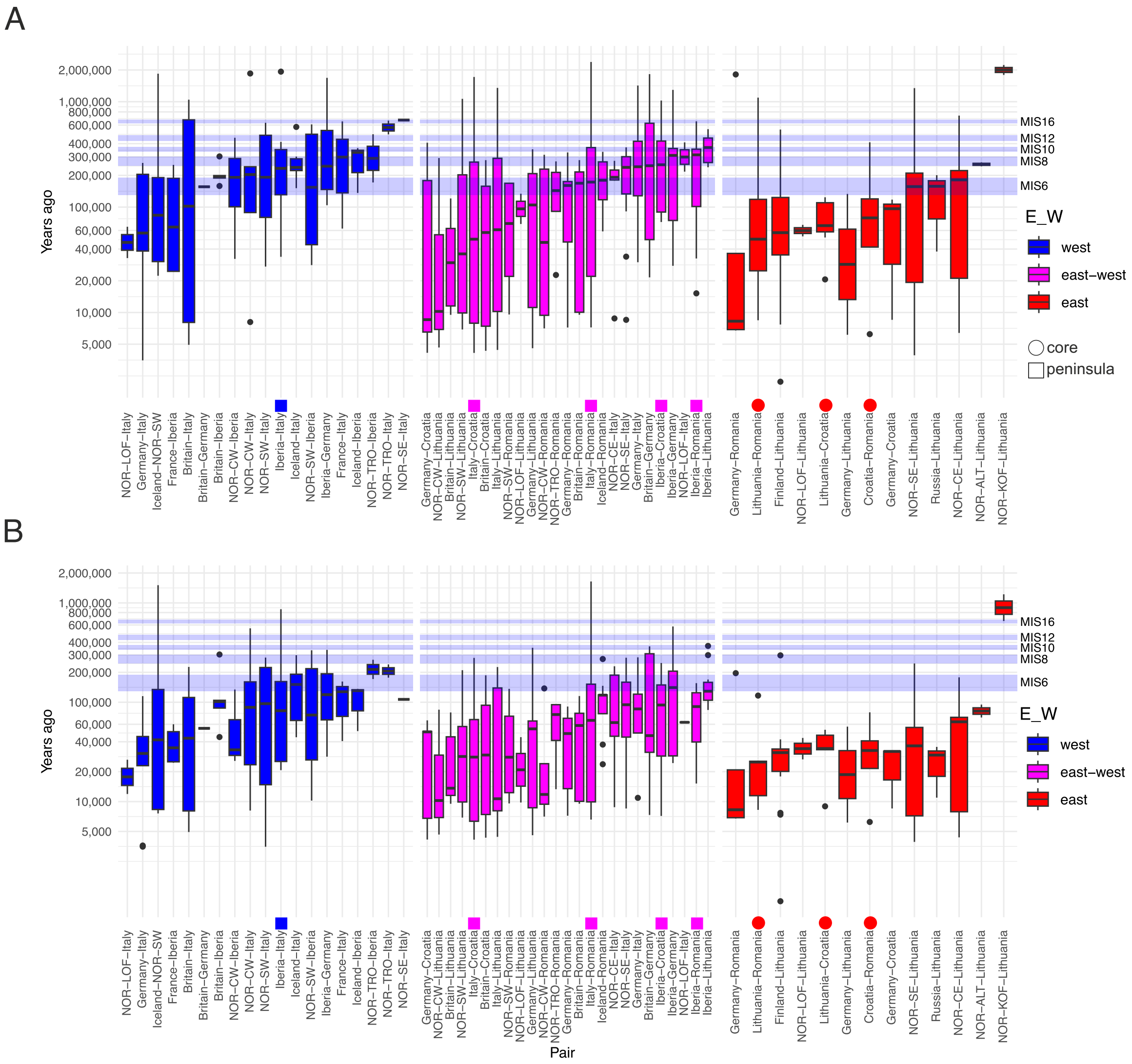

Supplementary Figure 15. Initial divergence (ya) between regional pairs of samples with A) (M<0.999) and B) (M<0.99). Circles after the sample pair names highlight initial divergence between core populations and rectangles between southern European Peninsulas. Glacial periods (MIS6-MIS16) shown by blue shading.

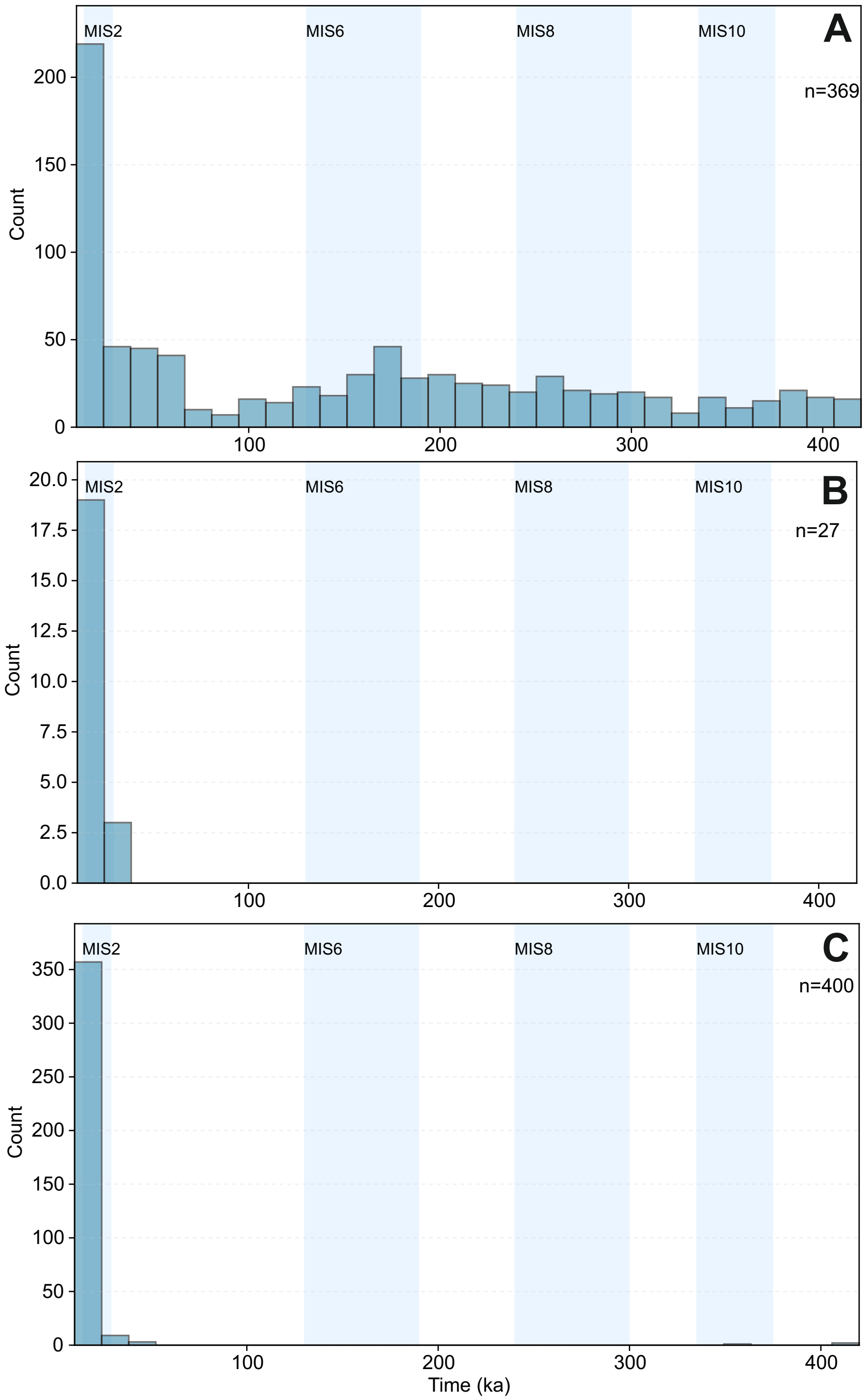

Supplementary Figure 16. Frequency of isolation event midpoints (time intervals where $m < 1{\times}10^{-7}$) through time (10–420 ka). (A) All sample comparisons. (B) Primary dataset showing the core pattern. (C) Bootstrap replicates exhibiting the core pattern. n=the number of runs included. MIS = Marine Isotope Stage; ka = thousand years ago.

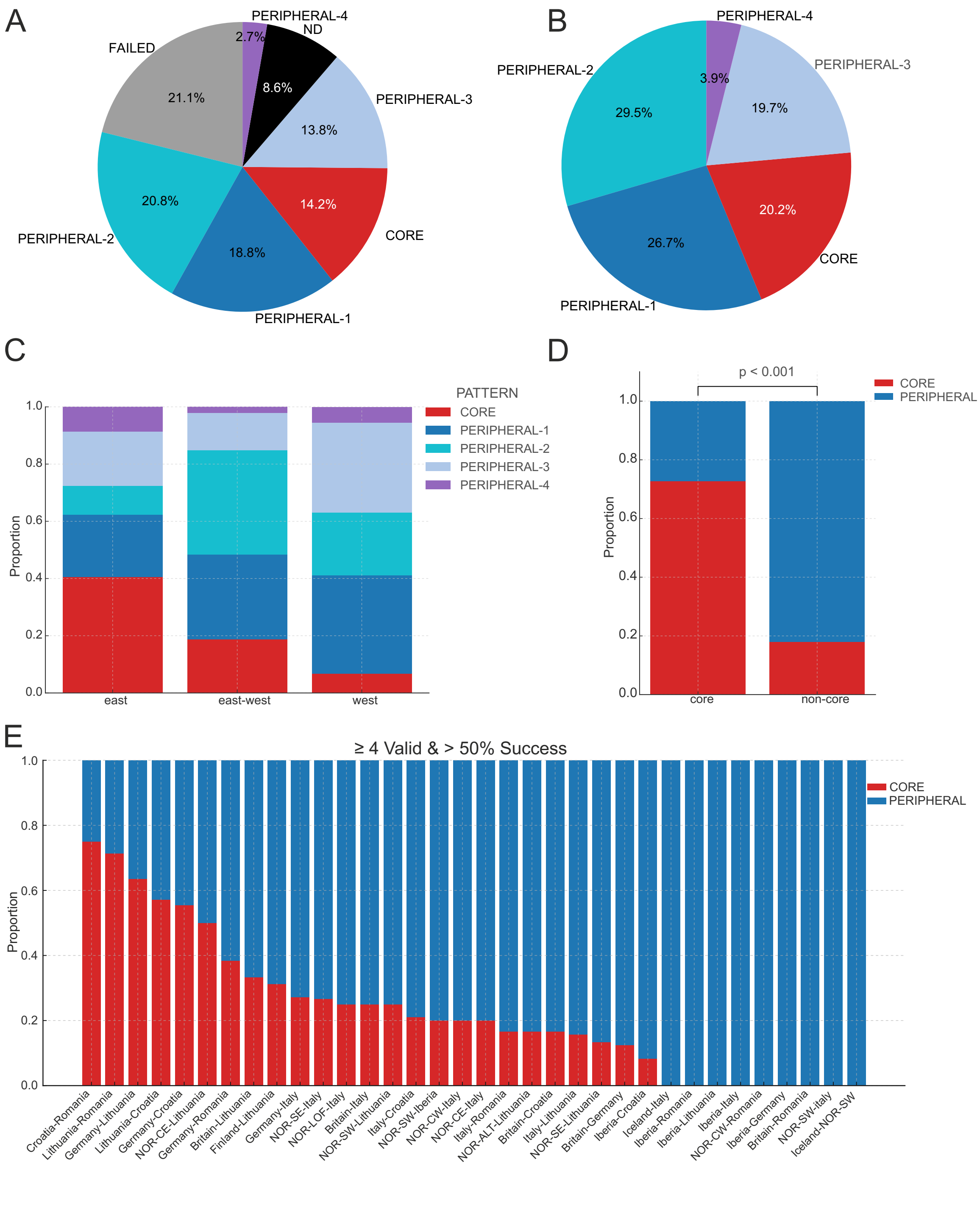

Supplementary Figure 17. (A) Proportions of different demographic history patterns across all sample comparisons. (B) Same as (A), but with FAILED and ND patterns excluded. (C) Proportions of different patterns in eastern, western, and east–west region pairs. (D) Core and peripheral (PERIPHERAL1–4) patterns in comparisons among core samples (Croatia–Romania, Lithuania–Croatia, and Lithuania–Romania haplotype combinations) and in all other comparisons (Non-core). Fisher's exact test was used to calculate the p-value for the difference. (E) Proportions of core and peripheral patterns across different regional pairs.

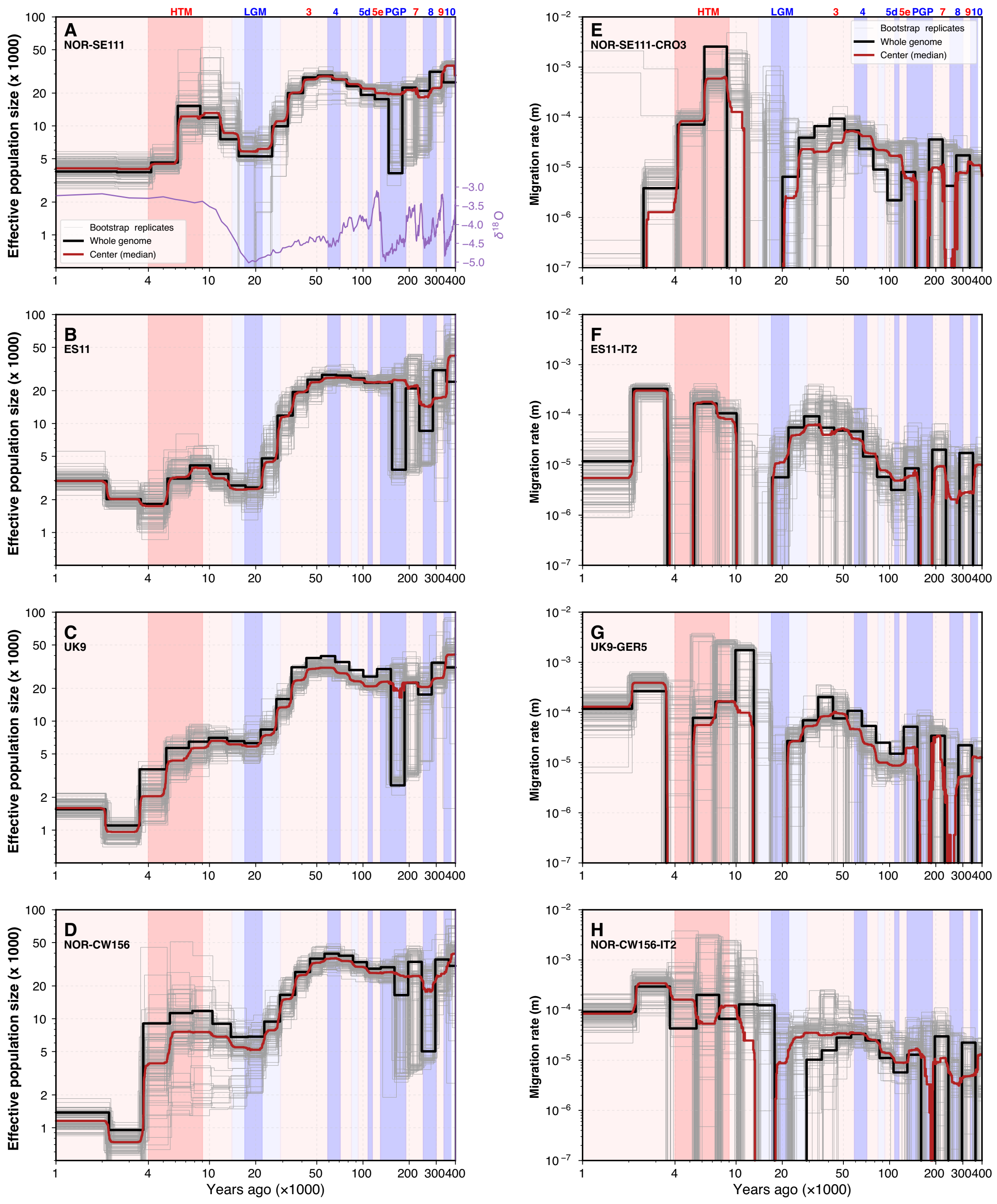

Supplementary Figure 18. Demographic history of the peripheral pattern with 100 bootstrap replicates. A-D) Effective population size of the shown samples. E-H) Migration rate between corresponding sample pairs. Red step line = bootstrap median (point-wise 50th percentile calculated as arithmetic median for effective population size and in logarithmic scale for migration rate). Black line = demographic trajectory of the sample inferred from whole-genome data. HTM=Holocene thermal maximum (9-4 ka ago, LGM=Last glacial maximum (22-17 ka ago), Numbers present different Marine Isotope Stages, even numbers showing glacial periods and

uneven numbers interglacial periods. A purple curve in panel A represent inverse benthic δ18O records from Lisiecki and Raymo (2005)[1] and serves as a proxy for historical temperature.

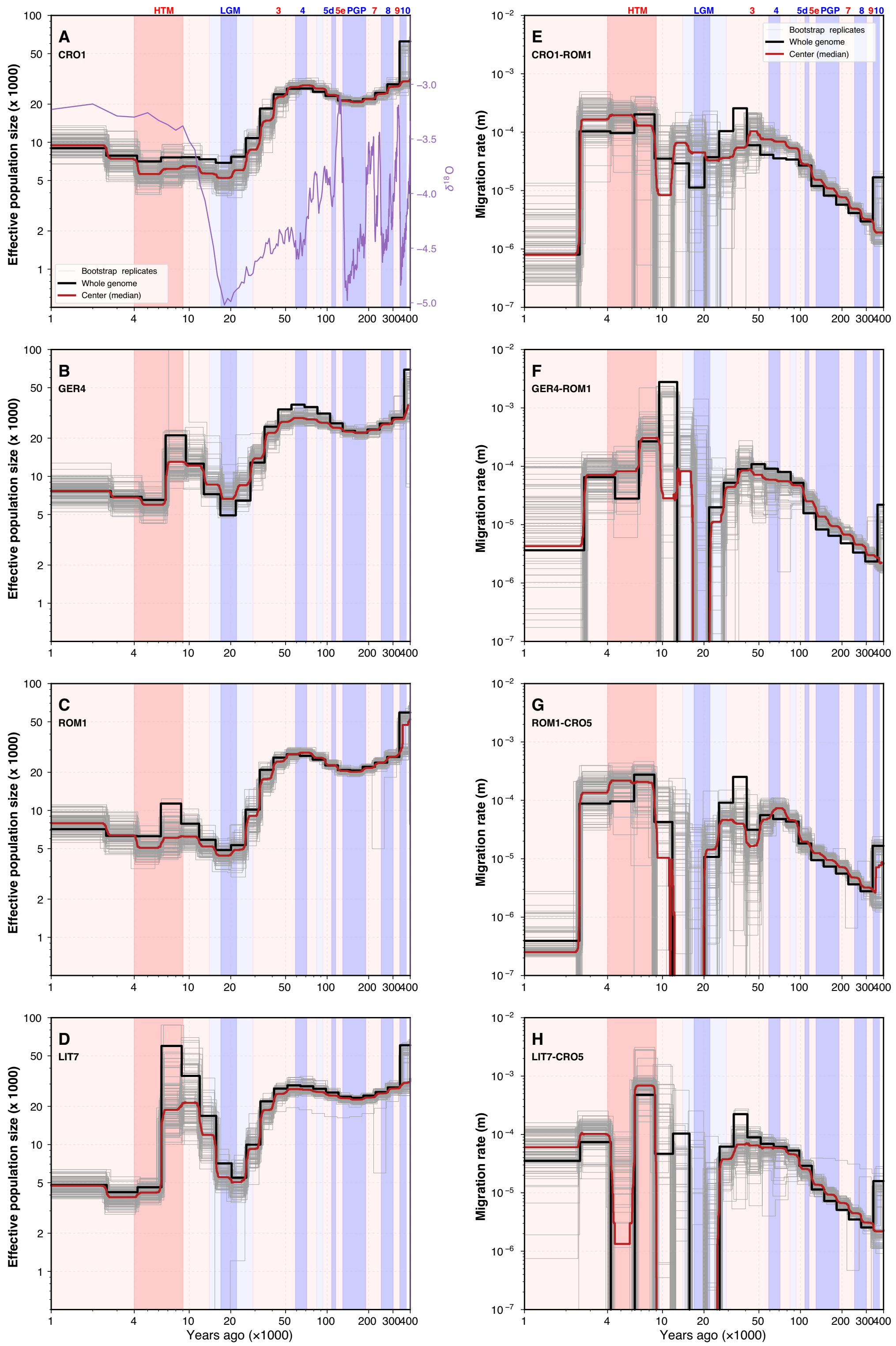

Supplementary Figure 19. Demographic history of the core pattern with 100 bootstrap replicates. A-D) Effective population size of the shown samples. E-H) Migration rate between corresponding sample pairs. Red step line = bootstrap median (point-wise 50th percentile calculated as arithmetic median for effective population size and in logarithmic scale for migration rate. Black line = demographic trajectory of the sample inferred from whole-genome data. HTM=Holocene thermal

maximum (9-4 ka ago, LGM=Last glacial maximum (22-17 ka ago). Numbers present different Marine Isotope Stages, even numbers showing glacial periods and uneven numbers interglacial periods. A purple curve in panel A represent inverse benthic δ18O records from Lisiecki and Raymo (2005)[1] and serves as a proxy for historical temperature.

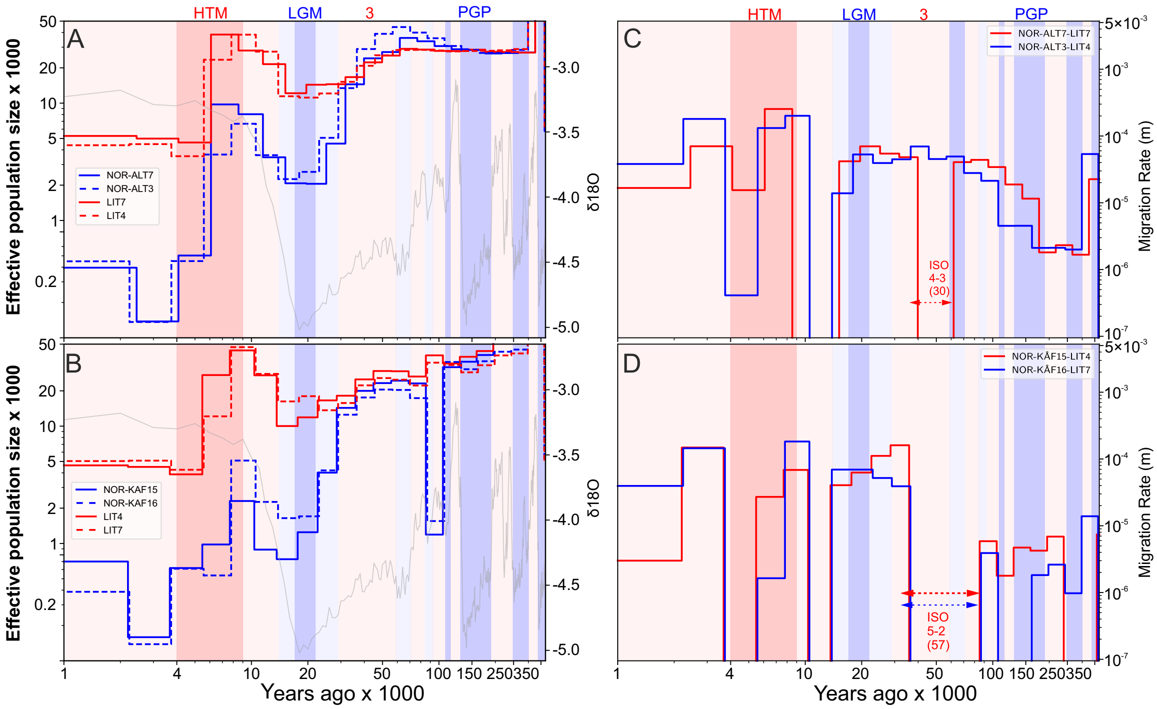

Supplementary Figure 20. Demographic histories of northern Norwegian Kåfjord and Alta populations. A) Effective population sizes of Kåfjord (blue) and Lithuania (red). B) Effective population sizes of Alta (blue) and Lithuania (red). C) Migration rate between Alta and Lithuania. D) Migration rate between Kåfjord and Lithuania. Glacial and interglacial periods are indicated by blue and red background shading, respectively, with numbers denoting specific Marine Isotope Stages (MIS). ISO = isolation event. Numbers in parentheses indicate the lengths of isolation events (in thousand years). HTM = Holocene Thermal Maximum (9,000–4,000 years ago); LGM = Last Glacial Maximum (22,000–17,000 years ago); PGP = Penultimate Glacial Period (190,000-130,000 years ago). The curve at the bottom of (A) and (B) represents the inverse benthic $\delta^{18}O$ record from Lisiecki and Raymo (2005)1, used as a proxy for historical temperature.ears ago).

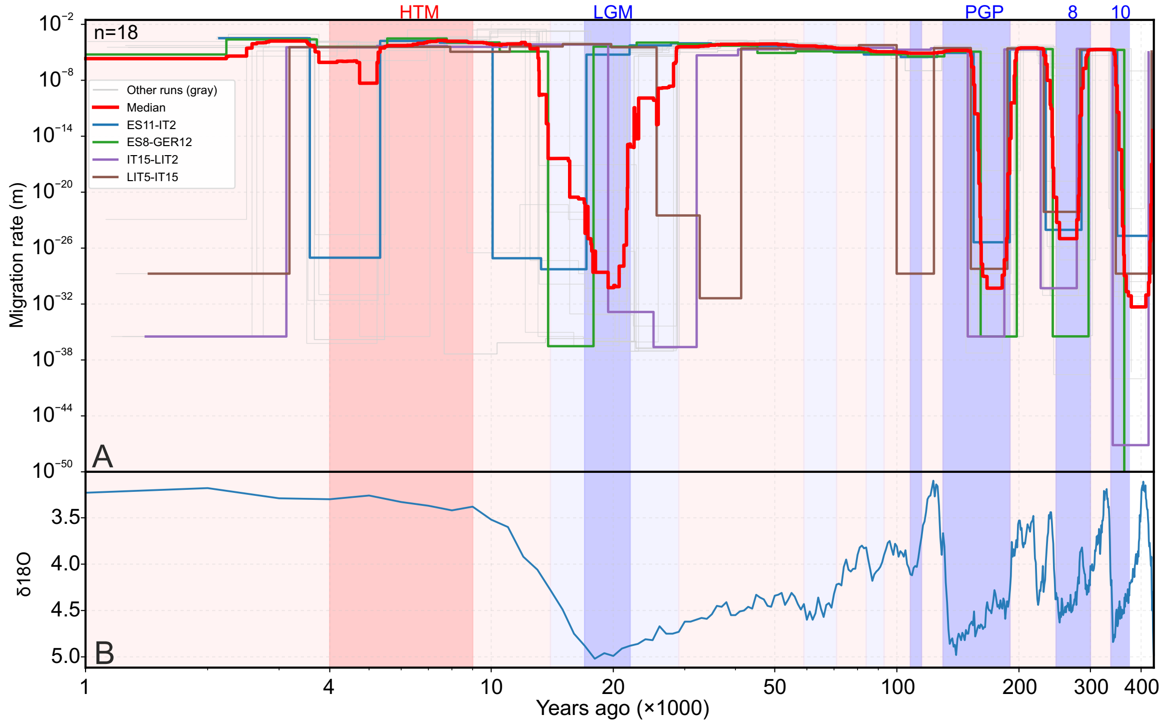

Supplementary Figure 21. Symmetric migration rate through time. A) peripheral patterns (PERIPHERAL 1) of history. The four most recent major glacial periods are shown (LGM=Last glacial maximum, PGP=Penultimate glacial period, 8=MIS 8, 10=MIS 10). HTM=Holocene thermal maximum. Individual runs drawn with colored lines where M<0.999 from MIS10 until present time. Red line - median migration rate across all runs (n=total number of runs). B) The δ18O isotope data from Lisiecki and Raymo (2005)[1]. Note that very low migration rate values are effectively zero, as seen between the western Italian (IT15) and eastern Lithuanian (LIT5, LIT2) populations today.

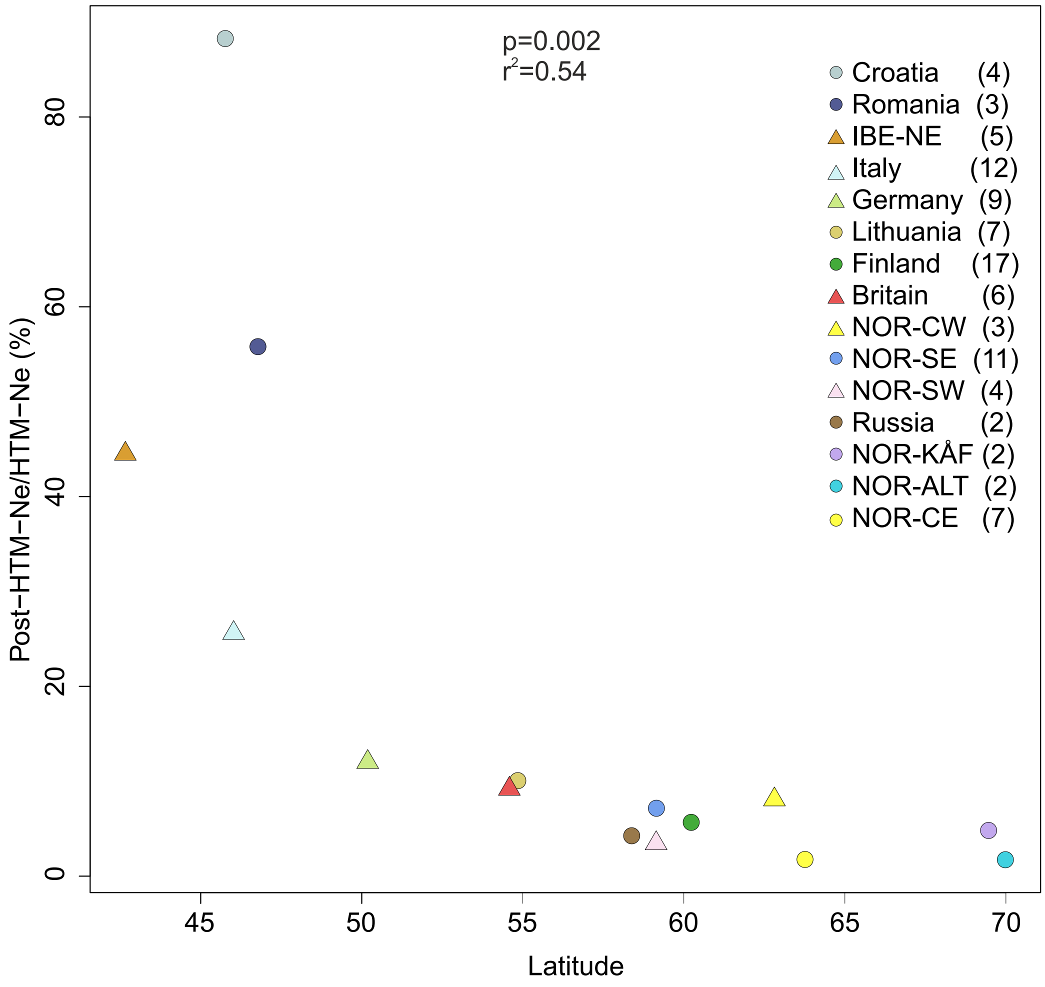

Supplementary Figure 22. Decline in effective population sizes after the Holocene Thermal Maximum (HTM). The y-axis shows the relative effective population size (%) after the HTM (post-HTM; 4000–2000 years ago) compared with peak values during the HTM. The Pearson correlation coefficient (r) and p-value were calculated using the cor.test function in R. Numbers in parentheses indicate the number of error-free runs used to estimate the ratio in each region, with triangles representing western regions and circles representing eastern regions.

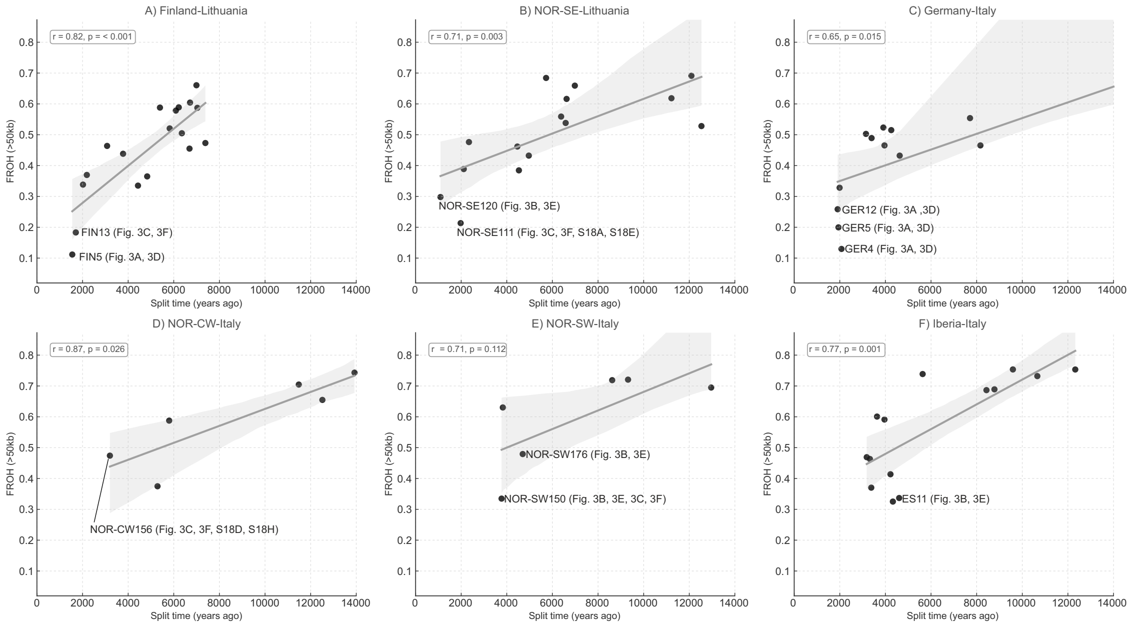

Supplementary Figure 23. Peripheral populations with the lowest inbreeding coefficients (hybrids) split most recently from their source populations. Correlation of split times and inbreeding coefficients (F_{ROH}). F_{ROH} values of samples of the first population mentioned in the figure title were plotted against the split time of the population pair. r=correlation coefficient, p=p-value based on cor.test function in R. Samples belonging to our primary dataset are highlighted in each figure panel.

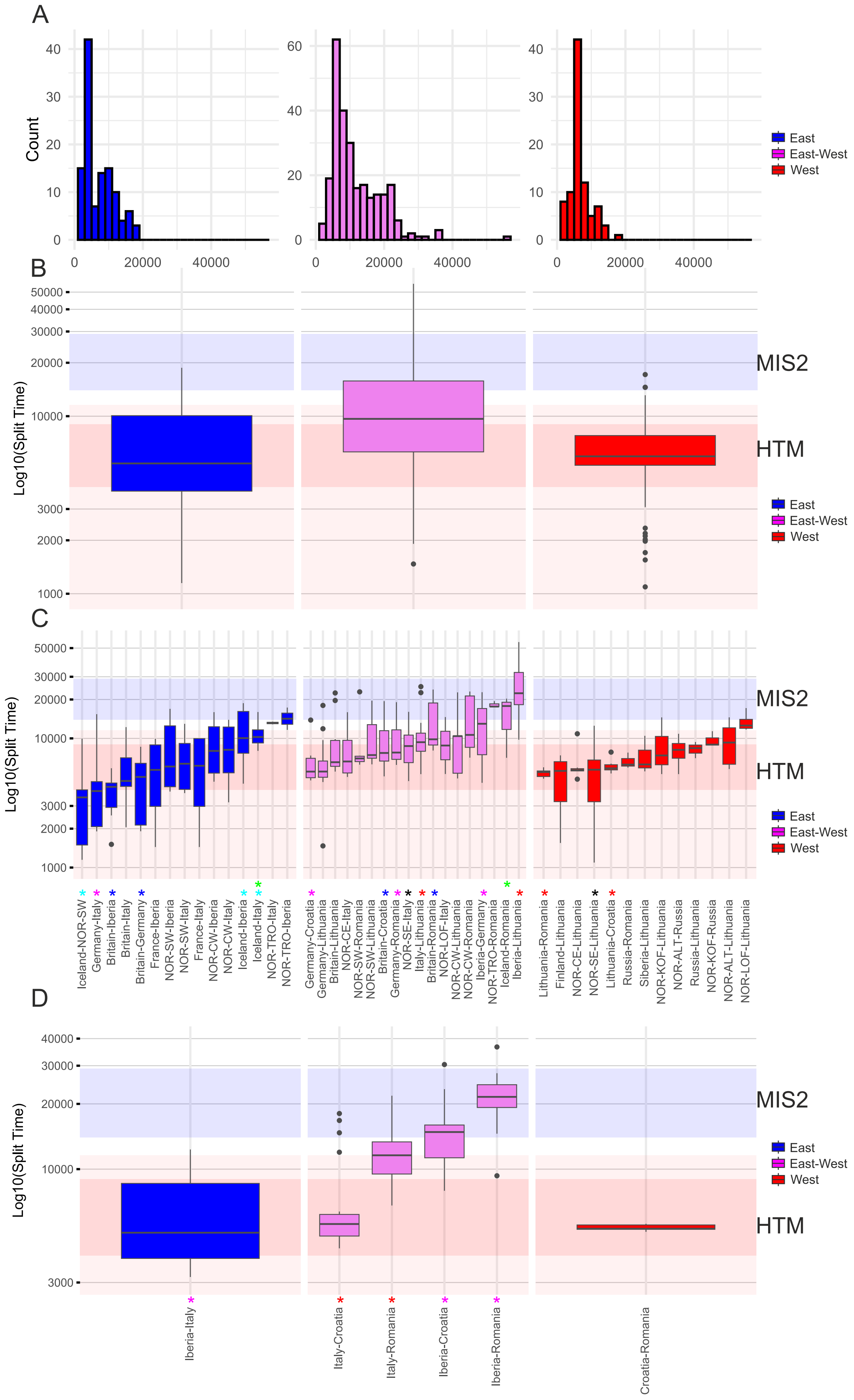

A
Count
East
East-West
West
B
Log10(Split Time)
MIS2
HTM
C
Iceland-NOR-SW
Germany-Italy
Britain-Iberia
Britain-Italy
Britain-Germany
France-Iberia
NOR-SW-Iberia
NOR-SW-Italy
France-Italy
NOR-CW-Iberia
NOR-CW-Italy
Iceland-Iberia
Iceland-Italy
NOR-TRO-Italy
NOR-TRO-Iberia
Germany-Croatia
Germany-Lithuania
Britain-Lithuania
NOR-CE-Italy
NOR-SW-Romania
NOR-SW-Lithuania
Britain-Croatia
Germany-Romania
NOR-SE-Italy
Italy-Lithuania
Britain-Romania
NOR-LOF-Italy
NOR-CW-Lithuania
NOR-CW-Romania
Iberia-Germany
NOR-TRO-Romania
Iceland-Romania
Iberia-Lithuania
Lithuania-Romania
Finland-Lithuania
NOR-CE-Lithuania
NOR-SE-Lithuania
Lithuania-Croatia
Russia-Romania
Siberia-Lithuania
NOR-KOF-Lithuania
NOR-ALT-Russia
Russia-Lithuania
NOR-KOF-Russia
NOR-ALT-Lithuania
NOR-LOF-Lithuania
D
Iberia-Italy
Italy-Croatia
Italy-Romania
Iberia-Croatia
Iberia-Romania
Croatia-Romania

Supplementary Figure 24. Comparison of split times (M < 0.5) between major groups (western, east–west, and eastern) and subgroups (between regions). A) Histograms of split times for major groups. B) Boxplots for the same three major groups, excluding samples from southern Europe (see D). C) Boxplots of split times for major groups and subgroups along the north–south axis. Colored stars at the bottom indicate significant differences in split times ($p < 0.05$) between western and eastern samples (Supplementary Data 7) for colonization of specific central or northern regions: red = Lithuania, blue = Britain, magenta = Germany, black = Norway-SE, green = Iceland. For colonization of Iceland (cyan stars), significant differences in split times were also observed within the western-origin samples. D) Split times of southern European populations (west–east direction). MIS2 = Marine Isotope Stage 2 (29–14 ka); Holocene (11.6–0 ka) is shaded in red; HTM = Holocene Thermal Maximum (9,000–4,000 years ago).

A

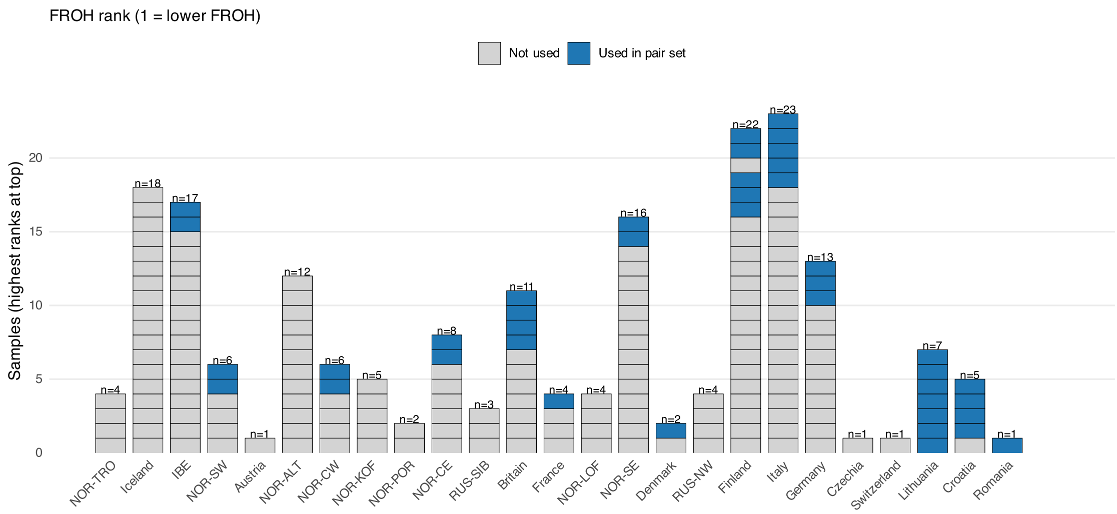
FROH rank (1 = lower FROH)
Not used
Used in pair set
Samples (highest ranks at top)
NOR-TRO
Iceland
IBE
NOR-SW
Austria
NOR-ALT
NOR-CW
NOR-KOF
NOR-POR
NOR-CE
RUS-SIB
Britain
France
NOR-LOF
NOR-SE
Denmark
RUS-NW
Finland
Italy
Germany
Czechia
Switzerland
Lithuania
Croatia
Romania

B

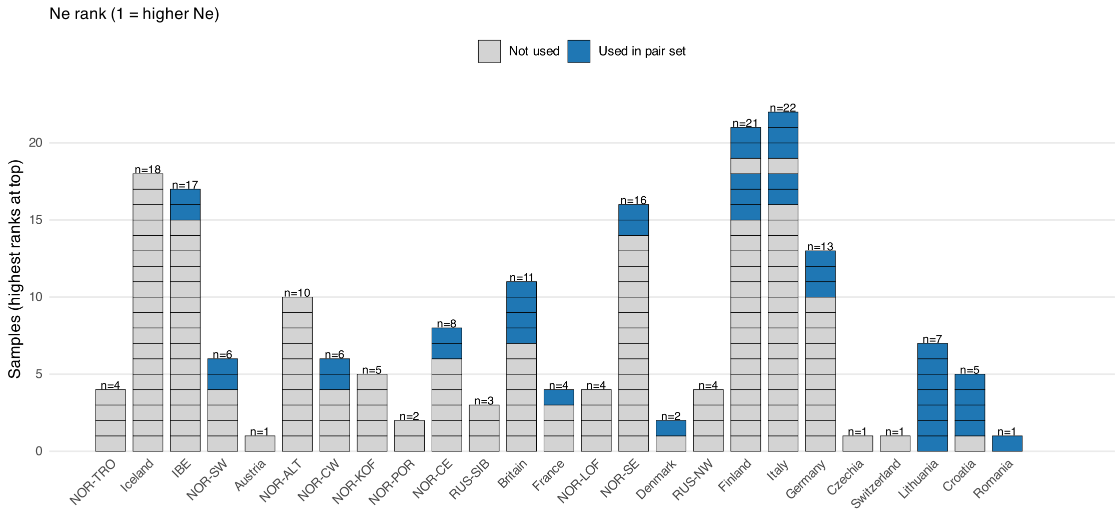
Ne rank (1 = higher Ne)
Not used
Used in pair set
Samples (highest ranks at top)
NOR-TRO
Iceland
IBE
NOR-SW
Austria
NOR-ALT
NOR-CW
NOR-KOF
NOR-POR
NOR-CE
RUS-SIB
Britain
France
NOR-LOF
NOR-SE
Denmark
RUS-NW
Finland
Italy
Germany
Czechia
Switzerland
Lithuania
Croatia
Romania

C

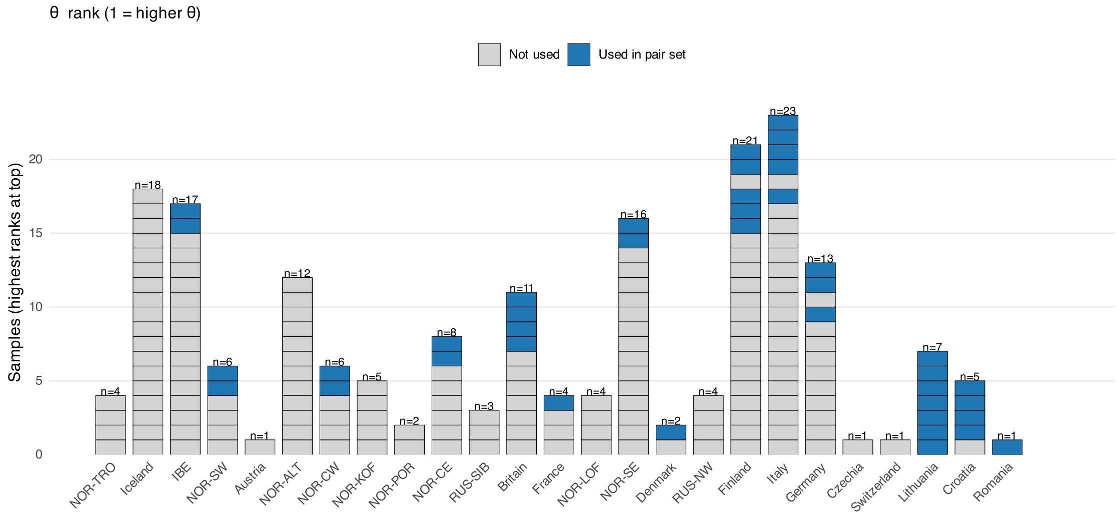
θ rank (1 = higher θ)
Not used
Used in pair set
Samples (highest ranks at top)
NOR-TRO
Iceland
IBE
NOR-SW
Austria
NOR-ALT
NOR-CW
NOR-KOF
NOR-POR
NOR-CE
RUS-SIB
Britain
France
NOR-LOF
NOR-SE
Denmark
RUS-NW
Finland
Italy
Germany
Czechia
Switzerland
Lithuania
Croatia
Romania

Supplementary Figure 25. Primary samples (N=41) selected for demographic inference from different regions. A-C) Samples ranked within regions based on their F_{ROH} (A), effective population size (B) and heterozygosity (C). Regions shown in x-axis are ordered by decreasing median F_{ROH} values. Abbreviations: IBE= Iberia, NOR-TRO=Norway-Tromsø, NOR-KÅF=Norway-Kåfjord, NOR-POR=Norway-Porsanger, NOR-ALT=Norway-Alta, NOR-CW=Norway-central-western, RUS-SIB=Russia-Siberia, Russia-NW=Russia-northwestern, Karelia=Russia-Karelia, NOR-SE=Norway-southeastern, NOR-LOF=Norway-Lofoten, NOR-SW=Norway-southwestern, NOR-CE=Norway-central-eastern.

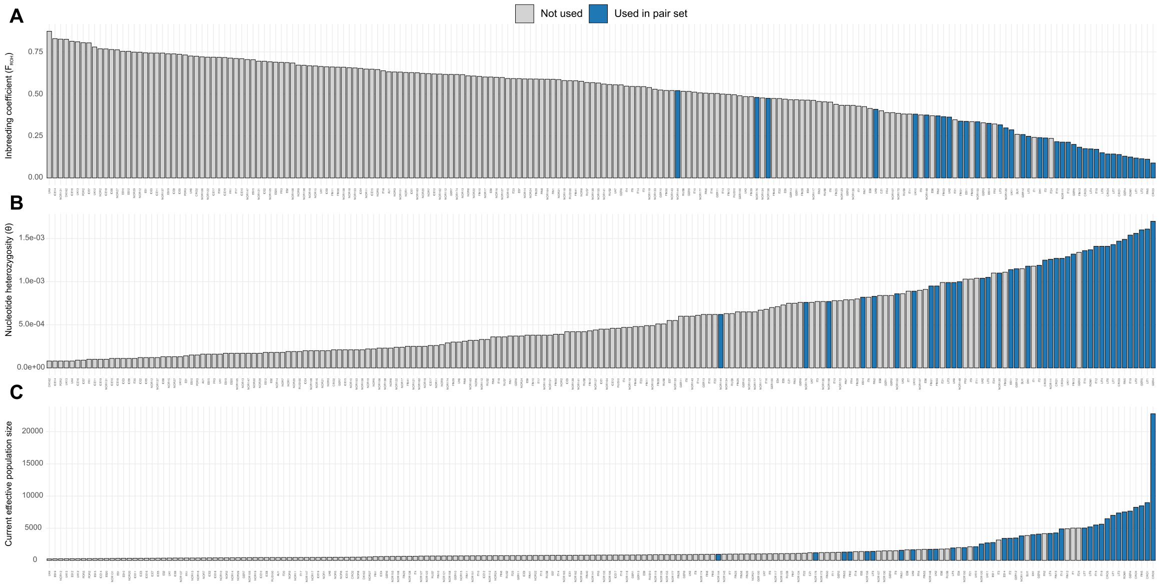

Supplementary Figure 26. Primary samples (N=41) shown in Fig. S25 for demographic inference across all samples. A-C) All samples ordered by F_{ROH} values (A), heterozygosity (B) and effective population size (C).

Supplementary Table 1. Genetic differentiation between regions (n=15). Weighted F_{ST} between 5 samples from each region is shown.

Region	N-KÅF	N-TRO	ICE	N-CW	N-CE	N-SW	N-SE	FIN	LIT	BRI	GER	CRO	IT	IBE-NE
N-ALT	0,77	0,69	0,76	0,58	0,53	0,62	0,52	0,40	0,45	0,62	0,52	0,54	0,61	0,74
N-KÅF		0,60	0,75	0,52	0,45	0,58	0,43	0,41	0,41	0,60	0,45	0,46	0,57	0,73
N-TRO			0,28	0,17	0,25	0,20	0,30	0,39	0,40	0,26	0,28	0,35	0,28	0,32
ICE				0,20	0,28	0,20	0,34	0,44	0,45	0,26	0,31	0,39	0,31	0,33
N-CW					0,04	0,03	0,09	0,22	0,24	0,08	0,09	0,18	0,12	0,21
N-CE						0,10	0,06	0,14	0,16	0,14	0,09	0,15	0,18	0,31
N-SW							0,14	0,28	0,29	0,11	0,14	0,23	0,16	0,26
N-SE								0,11	0,13	0,18	0,07	0,13	0,19	0,36
FIN									0,05	0,29	0,15	0,17	0,29	0,45
LIT										0,30	0,15	0,18	0,30	0,47
BRI											0,13	0,23	0,16	0,21
GER												0,10	0,11	0,26
CRO													0,18	0,36
IT														0,19

Abbreviations: N-ALT=Norway-Alta, N-KÅF=Norway-Kåfjord, N-TRO=Norway-Tromso, N-CW=Norway-centralwestern, N-CE=Norway-centraleastern, N-SW=Norway-southwestern,N-SE=Norway-southeastern, FIN=Finland, LIT=Lithuania, BRI=Britain, CRO=Croatia, IT=Italy, IBE-NE=Iberia-northeastern.

References:

1. Lisiecki LE, Raymo ME. 2005. A Pliocene-Pleistocene stack of 57 globally distributed benthic δ18O records. *Paleoceanography* 20: PA1003

2. Wang K, Mathieson I, O'Connell J, Schiffels S. 2020. Tracking human population structure through time from whole genome sequences. *PLOS Genetics* 16 (3): e1008552.
