# Supplementary material for: The Late Quaternary climate impact on the genome of the woodland strawberry (Fragaria vesca), a perennial herb
Source: Commun Biol. 2026 Jan 15;9:263. doi: 10.1038/s42003-026-09539-5 (PMC12913768; doi:10.1038/s42003-026-09539-5)
Supplement: Supplementary file 3 — Description of Additional Supplementary Files [file 42003_2026_9539_MOESM3_ESM.pdf]

Description of Additional Supplementary Files:

File name: Supplementary Data 1

Description: The sample information and sequencing statistics.

File name: Supplementary Data 2

Description: Source files for phylogenetic trees.

File name: Supplementary Data 3

Description: Source data for the admixture results, bioclimatic variables, effective population sizes and inbreeding coefficients.

File name: Supplementary Data 4

Description: Details of MSMC-IM runs and related statistical tests.

File name: Supplementary Data 5

Description: Source data for uncovering postglacial colonization patterns across Europe.
