# Supplementary material for: The Late Quaternary climate impact on the genome of the woodland strawberry (Fragaria vesca), a perennial herb
Source: Commun Biol. 2026 Jan 15;9:263. doi: 10.1038/s42003-026-09539-5 (PMC12913768; doi:10.1038/s42003-026-09539-5)
Supplement: Supplementary file 9 — Reporting summary [file 42003_2026_9539_MOESM9_ESM.pdf]

nature portfolio

Corresponding author(s): Prof. Timo Hytönen

Last updated by author(s): Dec 23, 2025

Reporting Summary

Nature Portfolio wishes to improve the reproducibility of the work that we publish. This form provides structure for consistency and transparency in reporting. For further information on Nature Portfolio policies, see our Editorial Policies and the Editorial Policy Checklist.

Statistics

For all statistical analyses, confirm that the following items are present in the figure legend, table legend, main text, or Methods section.

n/a	Confirmed	
☐	☒	The exact sample size (*n*) for each experimental group/condition, given as a discrete number and unit of measurement
☐	☒	A statement on whether measurements were taken from distinct samples or whether the same sample was measured repeatedly
☐	☒	The statistical test(s) used AND whether they are one- or two-sided<br>*Only common tests should be described solely by name; describe more complex techniques in the Methods section.*
☒	☐	A description of all covariates tested
☐	☒	A description of any assumptions or corrections, such as tests of normality and adjustment for multiple comparisons
☐	☒	A full description of the statistical parameters including central tendency (e.g. means) or other basic estimates (e.g. regression coefficient) AND variation (e.g. standard deviation) or associated estimates of uncertainty (e.g. confidence intervals)
☐	☒	For null hypothesis testing, the test statistic (e.g. *F*, *t*, *r*) with confidence intervals, effect sizes, degrees of freedom and *P* value noted<br>*Give P values as exact values whenever suitable.*
☒	☐	For Bayesian analysis, information on the choice of priors and Markov chain Monte Carlo settings
☒	☐	For hierarchical and complex designs, identification of the appropriate level for tests and full reporting of outcomes
☐	☒	Estimates of effect sizes (e.g. Cohen's *d*, Pearson's *r*), indicating how they were calculated

Our web collection on statistics for biologists contains articles on many of the points above.

Software and code

Policy information about availability of computer code

Data collection	Population structure was analyzed with SNPrelate (PCA), IQ-tree2 (SNP-phylogeny) and ADMIXTURE (admixture-analysis). Present and historical effective population sizes were analyzed with MSMC2 and MSMC-IM softwares. Inbreeding coefficients were collected using plink2 (FROH) and vcftools (Fis). Figures were generated using R and Python with custom scripts.
Data analysis	The code used for data-analysis is available at Github (https://github.com/tuomas64/strawberry) and Zenodo (https://doi.org/10.5281/zenodo.17990078).

For manuscripts utilizing custom algorithms or software that are central to the research but not yet described in published literature, software must be made available to editors and reviewers. We strongly encourage code deposition in a community repository (e.g. GitHub). See the Nature Portfolio guidelines for submitting code & software for further information.

Data

Policy information about availability of data

All manuscripts must include a data availability statement. This statement should provide the following information, where applicable:

- Accession codes, unique identifiers, or web links for publicly available datasets
- A description of any restrictions on data availability
- For clinical datasets or third party data, please ensure that the statement adheres to our policy

Genome sequence and GBS data have been deposited to NCBI (PRJNA1018297 and PRJNA1357314, respectively). All numerical data presented in this article have been deposited in the Dryad repository (https://doi.org/10.5061/dryad.8cz8w9h43).

Research involving human participants, their data, or biological material

Policy information about studies with human participants or human data. See also policy information about sex, gender (identity/presentation), and sexual orientation and race, ethnicity and racism.

Reporting on sex and gender	N/A
Reporting on race, ethnicity, or other socially relevant groupings	N/A
Population characteristics	N/A
Recruitment	N/A
Ethics oversight	N/A

Note that full information on the approval of the study protocol must also be provided in the manuscript.

Field-specific reporting

Please select the one below that is the best fit for your research. If you are not sure, read the appropriate sections before making your selection.

☐ Life sciences ☐ Behavioural & social sciences ☒ Ecological, evolutionary & environmental sciences

For a reference copy of the document with all sections, see nature.com/documents/nr-reporting-summary-flat.pdf

Ecological, evolutionary & environmental sciences study design

All studies must disclose on these points even when the disclosure is negative.

Study description	We sequenced 200 genomes to investigate the temporal dynamics of population structure in European woodland strawberry
Research sample	We collected 200 Fragaria vesca samples across Europe.
Sampling strategy	To maximize the geographic coverage of the 200 samples across Europe, we initially collected one individual per population. For more robust population-level analyses, our aim was to obtain at least five individuals per region. In selected regions, such as the Italian Alps, Finland, and Iceland, we collected approximately 20 individuals each to enable future investigations of potential natural selection. These analyses will be conducted in subsequent studies.
Data collection	Data was collected at the same time, when analyses were performed.
Timing and spatial scale	Samples have been collected before 2014. Data have been collected since 2015 and analyzed at the University of Helsinki, Finland
Data exclusions	We did not exclude any data, except a few outlier samples (recent hybrids). These samples have been mentioned/specified in the manuscript.
Reproducibility	All detailed steps to repeat data-analyses are described in Methods section.
Randomization	We randomized samples for calculating Fst between regions. We selected 5 random samples from each region if possible (some regions has less than 5 samples). For the block bootstrap, we randomly sampled 1-Mb blocks (30 × 1 Mb × 7 = 210 Mb) from the genome, approximating the full genome length (~220 Mb). Otherwise, we did not randomize data because we did not have that kind of experiments, which would have required randomizations.
Blinding	Blinding was not relevant for this study.

Did the study involve field work? ☐ Yes ☒ No

Reporting for specific materials, systems and methods

We require information from authors about some types of materials, experimental systems and methods used in many studies. Here, indicate whether each material, system or method listed is relevant to your study. If you are not sure if a list item applies to your research, read the appropriate section before selecting a response.

Materials & experimental systems

n/a	Involved in the study
☒	☐ Antibodies
☒	☐ Eukaryotic cell lines
☒	☐ Palaeontology and archaeology
☒	☐ Animals and other organisms
☒	☐ Clinical data
☒	☐ Dual use research of concern
☐	☒ Plants

Methods

n/a	Involved in the study
☒	☐ ChIP-seq
☒	☐ Flow cytometry
☒	☐ MRI-based neuroimaging

Plants

Seed stocks	Seeds are available for most genotypes with fully sequenced genomes. Resulting seedlings are not identical because original plants were not homozygote lines.
Novel plant genotypes	N/A
Authentication	N/A
